# Biomimetic MOF Nanocarrier‐Mediated Synergistic Delivery of Mitochondria and Anti‐Inflammatory miRNA to Alleviate Acute Lung Injury

**DOI:** 10.1002/advs.202416594

**Published:** 2025-02-25

**Authors:** Xin Shou, Changjiang Chen, Hangjie Ying, Zhiyun Liu, Lingyao Zeng, Qiujie Li, Lanjie Lei, Bingyong Mao, Wei Zhang, Shumao Cui, Liyun Shi

**Affiliations:** ^1^ Key lab of Artificial Organs and Computational Medicine Institute of Translational Medicine Zhejiang Shuren University Hangzhou Zhejiang 310015 China; ^2^ Department of Immunology Nanjing University of Chinese Medicine Nanjing Jiangsu 210023 China; ^3^ Department of Experiment Center Zhejiang Cancer Hospital Hangzhou Institute of Medicine (HIM) Chinese Academy of Sciences Hangzhou Zhejiang 310022 China; ^4^ State Key Laboratory of Food Science and Resources Jiangnan University Wuxi Jiangsu 214122 China

**Keywords:** acute lung injury, inflammation, macrophage, miRNA, MOF nanocarrier

## Abstract

Acute lung injury (ALI) is a clinically critical disease characterized by overwhelming inflammatory response and significant tissue damage with no specific treatment available currently. As a key player in the pathogenesis of ALI, macrophages are aberrantly activated and polarize toward the pro‐inflammatory phenotypes, leading to overzealous inflammation and lung injury. Mitochondria is recognized as a crucial signaling hub governing macrophage function and polarization, deregulation of which is causatively related with defective metabolism of macrophages, deregulated inflammation, and hence ALI. Herein, an inflammation‐responsive, biomimetic metal‐organic framework (MOF) nanoplatform, termed a127/mito@ZIF@Ma is developed, which is sophistically designed for synergistic delivery of macrophage‐derived mitochondria and anti‐inflammatory miRNA‐127 antagonist to resume pulmonary macrophages homeostasis and alleviate lung inflammation and injury. Notably, macrophage membrane encapsulation conferred the biomimetic MOF with enhanced transport efficacy both in vitro and in vivo. Therefore, the administration of the nanoparticles accordingly conferred a profound protection of mice against lung inflammation and injury induced by either bacterial or viral infection with unnoticeable tissue toxicity. The study thus devises a novel MOF‐based nanosystem that integrates mitochondria transplantation and miRNA therapeutics, which may open a new avenue for treating ALI and relevant critical diseases.

## Graphical abstract

A biomimetic MOF nanocarrier for macrophage‐targeting delivery of both mitochondria and anti‐miR‐127 is designed with an aim to revitalize macrophages and remold the inflammatory microenvironment for treating acute lung injury. By leveraging the macrophages‐targeting property and pH‐responsiveness, ZIF‐8 nanocarriers facilitated efficient delivery of anti‐inflammatory microRNAs and mitochondria, exerting the synergistic effects to rejuvenating the innate immune response and protect hosts from bacteria or virus‐induced ALI.

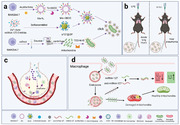

## 1 Introduction

Acute lung injury (ALI) is a type of respiratory disease characterized by uncontrolled lung inflammation and extensive tissue injury. Bacterial and viral infection are among the most common causes of ALI. Without timely and effective intervention, ALI progresses to loss of gas exchange, respiratory failure, and typically acute respiratory distress syndrome (ARDS) that is accompanied by high morbidity and mortality.^[^ ^1^, ^2^ ^]^ Although the mechanisms driving ALI and ARDS are not completely elucidated, accumulating evidences demonstrate that deregulated macrophages with metabolic and inflammatory distress contribute substantially to disease progression.

Macrophages are known as the first line of host defense, as well as a coordinator for the immune and inflammatory response. At the early stage of ALI, lung tissue resident macrophages, and monocyte‐derived macrophages develop into proinflammatory M1 macrophages, which secrete pro‐inflammatory cytokines, chemokines, and tissue‐damaging factors to cause more cells infiltration, the inflammation implication, and hence tissue injury.^[^ ^3^ ^]^ Under controlled inflammatory conditions, the proinflammtory macrophages undergo the phenotypic and functional transition and adopt the pro‐reparative M2 phenotype, which produce the anti‐inflammatory and pro‐resolving factors to promote the inflammation resolution and disease recovery.^[^ ^4^, ^5^ ^]^

Macrophage polarization is a fine‐tuning process that is orchestrated by a network of mechanisms including the metabolic and epigenetic program. Mitochondria is a central signaling hub that plays a central role in metabolic regulation of macrophage function and polarization. Dysfunctional or damaged mitochondria prove to cause impaired oxidative phosphorylation (OXPHOS), defective ATP synthesis, and glycolysis‐biased metabolism, leading to macrophages with insufficient bioenergetics, inflammatory phenotype, and compromised regenerative capability.^[^ ^6^, ^7^ ^]^ Thus, reinvigorating mitochondria biogenesis or delivering exogenous mitochondria has become an attractive approach to promote metabolism homeostasis and pro‐regenerative macrophages polarization for alleviating ALI and relevant diseases.^[^ ^8^, ^9^ ^]^ Recent studies, by us and other investigators, demonstrated the promising outcome of mitochondrial transplantation in treating the inflammatory and degenerative diseases, sparking the increase interest in harnessing this process for therapeutic purposes.^[^ ^10^, ^11^ ^]^ It has been reported that mitochondria can be transferred between cells through transient cellular connections, extracellular vesicle‐mediated delivery or tunneling nanotubes‐based transportation, etc.^[^ ^12^, ^13^, ^14^ ^]^ However, several obstacles such as limited efficiency, non‐cell specificity, and lower biological stability hinder the application of current mitochondrial transplantation as a therapeutic approach.^[^ ^15^ ^]^ The development of an efficient, viable, and safe mitochondrial delivery system is urgently needed.

In addition to metabolic regulatory machinery, genetic and epigenetic pathways exert the essential roles in determining macrophage cell fate and disease manifestation. MicroRNAs (miRNAs), the small non‐coding RNAs capable of post‐transcriptionally regulating target genes, are critically involved in the pathogenesis of ALI and ARDS and hence identified as the biomarkers and potential therapeutic targets.^[^ ^16^, ^17^ ^]^ In particular, miR‐127 has been reported to have a crucial role in promoting lung inflammation and injury, and its serum level correlates with disease severity.^[^ ^18^, ^19^ ^]^ Our previous study revealed that miR‐127 directly targeted B‐cell lymphoma 6 (Bcl6) and dual‐specificity phosphatase 1 (Dusp 1), and promoted differentiation of M1 pro‐inflammatory macrophages via c‐Jun N‐terminal kinase (JNK) pathway.^[^ ^20^ ^]^ Of interest, recent studies indicate that miR‐127 also exerts the negative effects on mitochondrial activity, and represses mitochondrial metabolism relevant to diseased conditions.^[^ ^21^, ^22^ ^]^ The data thus point to miR‐127 as a key signaling nexus that simultaneously modulates the inflammatory and metabolic pathways, and antagonizing this pathway (e.g., by applying anti‐miR‐127) is highly desirable to correct pulmonary macrophages with a dual mechanism of action.

**Figure 1:**
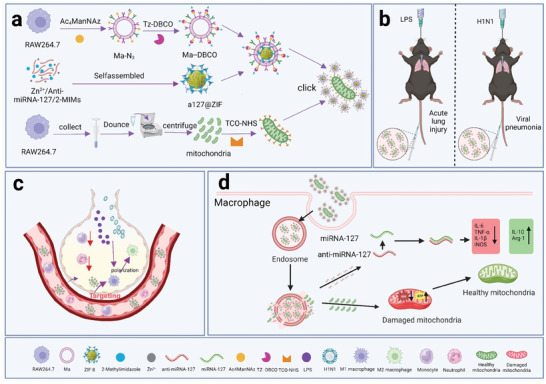
Schematic diagram of the design and synthesis of a127/mito@ZIF@Ma and its function. a) The synthetic procedures of the a127/mito@ZIF@Ma. b) a127/mito@ZIF@Ma is administered through the tail vein for the treatment of acute lung injury and viral pneumonia. c) The nanoparticles targets and regulates the function of damaged macrophages in the lungs, reducing the ratio of neutrophils to monocytes during acute lung injury. d) The nanoparticles are designed to release anti‐miRNA‐127 and mitochondria components in response to an acidic environment, thereby facilitating therapeutic interventions for acute lung injury or viral pneumonia therapy. The schematic diagram was generated using BioRender under a licensed subscription (Agreement number: JW27WEQPW9).

With increasing appreciation of the pathophysiological roles of miRNAs, the enthusiasm for the development of miRNA‐based therapeutics has been more inspired.^[^ ^23^, ^24^, ^25^ ^]^ However, the challenges, such as its vulnerability to in vivo microenvironment, unsuccessful delivering efficacy, rapid clearance by nucleases, and potential tissue toxicity still remain.^[^ ^26^, ^27^ ^]^ In an effort to identify the ideal delivery system for both mitochondria and miRNA therapeutics, we exploit the nano‐metal organic frameworks (MOF) because of their high loading capacity, stable and on‐demand release, and good biocompatibility and safety.^[^ ^23^, ^28^, ^29^ ^]^ Specifically, we herein developed a biomimetic MOF nanocarrier for macrophage‐targeting delivery of both mitochondria and anti‐miR‐127, with an aim to revitalize macrophages and remold the inflammatory microenvironment for treating acute lung injury (**Figure** **1**). For this, anti‐miR‐127 were loaded into ZIF‐8 nanoparticles and camouflaged by macrophage‐derived cell membranes, which were then conjugated with healthy mitochondria by a bio‐orthogonal chemistry strategy. By leveraging the macrophages‐targeting property and pH‐responsiveness, ZIF‐8 nanocarriers facilitated efficient delivery of anti‐inflammatory microRNAs and mitochondria, exerting the synergistic effects to rejuvenating the innate immune response and protect hosts from bacteria or virus‐induced ALI.

## 2 Results

### 2.1 Preparation and Characterization of a127/mito@ZIF@Ma Nanoparticles

In a typical experiment, one‐pot synthesis approach was employed to produce ZIF‐8 nanoparticles and encapsulate anti‐miRNA‐127 within these nanoparticles. This synthesis was achieved by pre‐mixed anti‐miRNA‐127 with a zinc ion solution, followed by the addition of 2‐methylimidazole to initiate the self‐assembly process, resulting in the formation of anti‐miRNA‐127@ZIF‐8 nanoparticles, hereafter referred as a127@ZIF. In order to improve the biocompatibility of a127@ZIF and endow those nanoparticles with macrophage‐targeting capability, cell membranes derived from RAW264.7 cells were purified and subsequently applied to the surface modification of a127@ZIF nanoparticles through ultrasound and extrusion techniques, resulting in the formation of biomimetic nanoparticles, referred as a127@ZIF@Ma.

In order to conjugate above nanoparticles with mitochondria, a methodology that integrates metabolic glucose engineering and bioorthogonal chemistry was utilized, as previously reported.^[^ ^30^, ^31^, ^32^ ^]^ RAW264.7 cells were treated with Ac4ManNAz to incorporate azide groups into transmembrane proteins. These azide groups subsequently reacted with TZ‐DBCO, resulting in the grafting of TZ groups onto the cell membrane. At the same time, mitochondria were freshly isolated from RAW264.7 cells and reactive with TCO‐NHS. The TCO‐modified mitochondria were then incubated with TZ‐labeled a127@ZIF@Ma, leading to the formation of the final formulates, a127/mito@ZIF@Ma.

**Figure 2:**
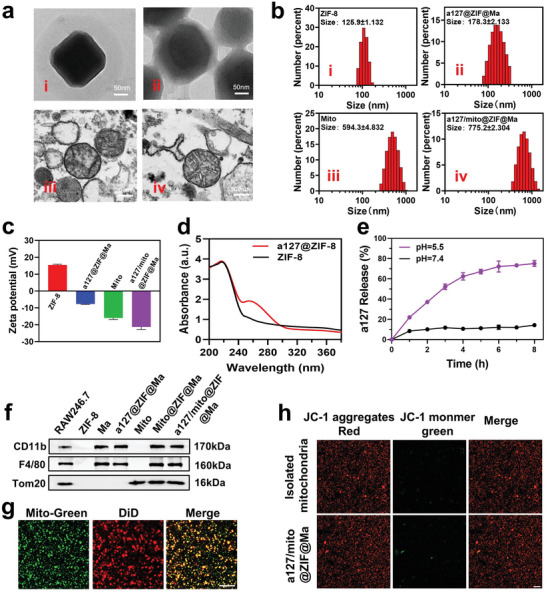
Preparation and characterization of a127/mito@ZIF@Ma NPs. (a) TEM images of ZIF‐8, a127@ZIF@Ma, Mito (Mitochondria), and a127/mito@ZIF@Ma NPs. (b,c) Particle size (b) and ζ‐potential (c) of ZIF‐8, a127@ZIF@Ma, Mito, and a127/mito@ZIF@Ma NPs. (d) UV–vis absorbance spectra for ZIF‐8 and a127@ZIF NPs. (e) Release curves of anti‐miRNA‐127 from a127@ZIF@Ma NPs at pH 7.4 and 5.5 over time. The data were represented as mean ± SD (*n* = 3). (f) Western blot analysis of the protein expression of F4/80, CD11b, and Tom20 in RAW264.7 cells, ZIF‐8, Ma, a127@ZIF@Ma, Mito, Mito@ZIF@Ma, and a127/mito@ZIF@Ma, respectively. (g) The coupling of mitochondria and a127 @ZIF@Ma using an orthogonal reaction. Mitochondria was labeled by Mito‐tracker Green and cell membrane was labeled by DiD. Scale bar, 10 µm. (h) Representative confocal images of JC1‐stained mitochondria and a127/mito@ZIF@Ma. Scale bar, 20 µm.

The morphology and particle diameter of ZIF‐8, a127@ZIF@Ma, and a127/mito@ZIF@Ma was characterized using transmission electron microscopy (TEM). As illustrated in **Figure** **2**a,b, ZIF‐8 had a monodisperse and uniform size distribution, with an average diameter of ≈125.9 ± 1.132 nm. In contrast to ZIF‐8, a visible membrane was successfully observed on the surface of a127@ZIF@Ma nanoparticles, which resulting in an increase of particle size to 178.3 ± 2.133 nm. Furthermore, the average particle size of the isolated mitochondria was 594.3 ± 4.832 nm, while the particle size of a127/mito@ZIF@Ma was 775.2 ± 2.304 nm. In addition, zeta potential of ZIF‐8, a127@ZIF@Ma, mitochondria, and a127/mito@ZIF@Ma NPs were further determined by electrophoretic light scattering (ELS). Due to the presence of negative head groups in phospholipids, phospholipid bilayer of cell membrane exhibited a significant negative charge. Hence, the zeta potential of nanoparticles changed from 15 to −8 mV, confirming that cell membranes were successfully coated to the surface of a127@ZIF. After conjugated with mitochondria, the zeta potential of a127/mito@ZIF@Ma further decreased from −16 to −20 mV, confirming the successful coupling of mitochondria with a127 @ZIF@Ma NPs (Figure 2c). To validate the encapsulate efficiency (EE) of anti‐miRNA‐127 within ZIF‐8 nanoparticles, the characteristic absorption of nuclear acid at 260 nm was examined through a UV absorption spectroscopy. A significant absorption was found in a127@ZIF nanoparticles, which was absent in ZIF‐8, demonstrating that anti‐miRNA‐127 was successfully encapsulated within ZIF‐8 nanoparticles (Figure 2d).

Due to the non‐covalent bond between Zn^2+^ and 2‐methylimidazole, ZIF‐8 nanoparticles possessed the pH‐sensitive and biodegradable properties in acid environment. As shown in Figure 2e, a slow release of anti‐miRNA‐127 was presented in physiological conditions (pH 7.4), while the release was accelerated in acidic environment (pH 5.5), which may attribute to the dissociation of the zinc‐imidazole coordination bond. Based on the calibration curve shown in Figure S1a (Supporting Information), the encapsulation efficiency of anti‐miR‐127 was calculated to be 91.40%, and the drug loading capacity (DLC) was ≈4.0 wt.%.

To further confirm the successful functionalization of a127/mito@ZIF@Ma NPs, membrane protein expression of RAW264.7 cells lysates and membrane proteins coated on a127/mito@ZIF@Ma NPs were compared and analyzed by SDS‐PAGE. The result showed that our membrane coating approaches by physical ultrasound and extrusion were able to retain most of the macrophage‐derived membrane proteins when constructing a127/mito@ZIF@Ma nanoparticles (Figure 2f; Figure S1b, Supporting Information). In addition, the higher expression of macrophage surface protein F4/80 and CD11b on a127/mito@ZIF@Ma NPs also demonstrated the coating success, which endowed the nanoparticles with macrophage homologous targeting ability (Figure 2f). Furthermore, co‐localization of the mitotracker Green‐labeled mitochondria with the DID‐labelled cell membrane was observed in the a127/mito@ZIF@Ma NPs, demonstrating the successful coupling of the mitochondria and a127@ZIF@Ma (Figure 2g). The treatment with Tz‐DBCO or TCO‐NHS had little effect on cell viability at concentrations up to 100 µm, as described before. Therefore, JC‐1 staining was employed to detect the bioenergetic potential of mitochondria. In freshly isolated mitochondria and mitochondria‐conjugated a127@ZIF@Ma NPs, JC‐1 predominantly presented as JC‐1 aggregates (red, Figure 2h). However, mitochondria that have undergone freeze‐thaw action exhibit a decrease in membrane potential and form JC‐1 monomers (green, Figure S2, Supporting Information). Therefore, the mitochondrial membrane potential of a127/mito@ZIF@Ma NPs was not significantly reduced, indicating that the biological activity of mitochondria was not affected after bioorthogonal chemistry.

### 2.2 Cellular Uptake and Lysosomal Escape of a127/mito@ZIF@Ma NPs

Next, we investigated the in vitro cytotoxic effects of ZIF‐8 NPs on RAW264.7 cells. Following a 48‐h incubation, the cell viability was decrease followed with the increase of the nanoparticle concentration. Since high concentrations of ZIF‐8 can significantly increase intracellular Zn^2+^ levels, subsequently causing an increase in ROS. As a result, the cell viability showed an abrupt decrease when the nanoparticle concentrations exceeded 40 µg mL^−1^. It was found that cell viability ratio was ≈88.7% when adopted with 30 µg mL^−1^ ZIF‐8 NPs, indicating a favorable biocompatible concentration under 30 µg mL^−1^ (Figure S3a, Supporting Information). Therefore, a concentration of 30 µg mL^−1^ a127@ZIF@Ma NP was selected as the optimal condition for subsequent experiments. We then conjugated a127 @ZIF@Ma NPs (30 µg mL^−1^) with varying concentrations of mitochondria and assessed their impact on the proliferation of RAW264.7 cells. As shown in Figure S3b (Supporting Information), cell proliferation was not affected within a specific range (0–40 µg mL^−1^, µg of protein). Nevertheless, exposure to mitochondrial concentrations exceeding 40 µg mL^−1^ resulted in a significant reduction in cell viability (Figure S3b, Supporting Information). Based on the above results, a dose of 15 µg mL^−1^ mitochondria was chosen to conjugated with a127@ZIF@Ma NPs.

**Figure 3:**
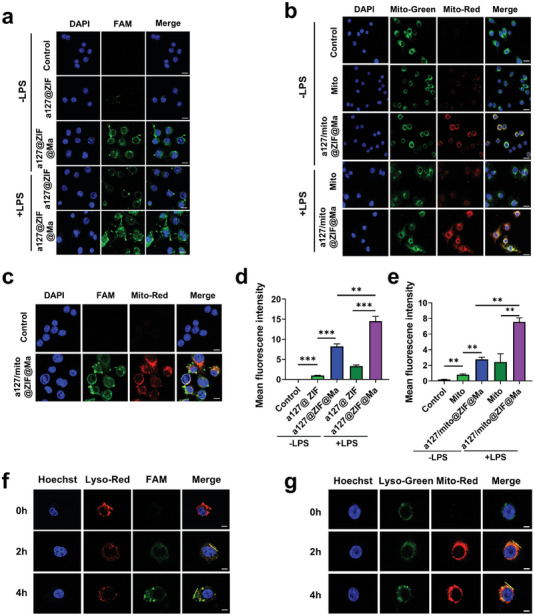
Cellular uptake and lysosomal escape of FAM‐a127@ZIF@Ma NPs. (a,d) Confocal fluorescence images of RAW264.7 cells treated with either FAM‐a127@ZIF NPs or FAM‐a127@ZIF@Ma NPs (30 µg mL^−1^) for 12 h, with or without the stimulation of LPS (100 ng mL^−1^). Scale bar, 25 µm. (b,e) Confocal fluorescence images of RAW264.7 cells treated with Mito or a127/Mito@ZIF@Ma NPs (15 µg mL^−1^, µg of protein) for 12 h, with or without the stimulation of LPS (100 ng mL^−1^). Exogenous mitochondria were labeled with mito‐tracker red, while endogenous mitochondria were labeled with mito‐tracker green. Scale bar, 25 µm. (c) The release of anti‐miRNA‐127 and mitochondria into the cytoplasm after the co‐incubation of a127/mito@ZIF@Ma NPs with RAW264.7 cells for 12 h. Scale bar, 10 µm. (f) The co‐localization of a127/mito@ZIF@Ma and lysosomes after 2‐ and 4‐h incubation. Anti‐miRNA‐127 was labeled with FAM, and lysosomes were visualized using lysoTracker red. Scale bar, 10 µm. (g) The co‐localization of a127/mito@ZIF@Ma and lysosomes after 2 and 4‐h incubation. Mitochondria was labeled with Mitotracker Red, and lysosomes were visualized using lysoTracker green. Scale bar, 10 µm. All the data are expressed as the mean ± SD (*n* = 3); **p < 0.01, ***p < 0.001.

In order to track the cellular uptake process, a green fluorescent dye 6‐carboxyfluorescein (FAM) was conjugated with anti‐miRNA‐127 and subsequently encapsulated into ZIF‐8 nanoparticles to prepare FAM‐a127@ZIF NPs and FAM‐a127@ZIF@Ma NPs. These nanoparticles were co‐incubated with RAW264.7 cells for 12 h, followed by treatment with or without lipopolysaccharide (LPS, 100 ng mL^−1^) for 12 h. The efficiency of cellular uptake by RAW264.7 cells in both normal and inflammatory conditions was assessed using confocal fluorescence microscopy. As shown in **Figure** **3**a, an obvious green fluorescence was observed when cells were treated with a127@ZIF@Ma NPs, whereas the cells treated with a127@ZIF NPs exhibited negligible fluorescence. This result suggested that cell membrane coated on the surface of a127@ZIF NPs enhanced the uptake progress. Furthermore, a comparison between LPS‐treated and non‐treated groups was carried out, and the results revealed that an increased green fluorescence following LPS treatment, which may be due to inflammation promoting the endocytosis of nanoparticles by RAW264.7 cells (Figure 3a,d). To further evaluate the uptake of a127/mito@ZIF@Ma, mitochondria were pre‐labeled with mitotracker Red, and conjugated with a127@ZIF@Ma to create a127/mito@ZIF@Ma nanoparticles. The same treatment protocol was applied, and the cellular uptake of free mitochondria versus a127/mito@ZIF@Ma NPs was evaluated. The results showed that ZIF@Ma NPs significantly enhanced the mitochondrial uptake efficiency compared to free mitochondria, potentially due to the internalization of membrane‐coated ZIF. Additionally, the uptake of mitochondria was also found to be elevated following LPS treatment (Figure 3b,e). In addition, we observed that FAM‐anti‐miRNA‐127 and mitotracker Red labeled mitochondria were successfully taken up by cells and released into the cytoplasm (Figure 3c).

To further explore the release process of a127/mito@ZIF@Ma NPs, FAM‐labeled a127/mito@ZIF@Ma NPs were cultured with RAW264.7 cells and a specific probe (Lysotracker Red) was used to examined the colocation of lysosomes with distribution of anti‐miRNA‐127 in cells at different time points. An abundant green fluorescence (FAM) was observed at 2 h, and most of the green fluorescence overlapped with the red fluorescence (Lysotracker Red), while there was only a small amount of overlap at 4 h (Figure 3f; Figure S4a, Supporting Information). Meanwhile, a127/mito@ZIF@Ma NPs were labeled with mitotracker Red and incubated with RAW264.7 cells for the indicated time, and the lysosome probe (Lysotracker Green) was used to label the lysosomes of RAW264.7 cells. The results showed that abundant red fluorescence (mitotracker Red) was observed at 2 h and mostly overlapped with green fluorescence (Lysotracker Green), while only a small amount overlapped at 4 h (Figure 3g; Figure S4b, Supporting Information). These results indicate that a127/mito@ZIF@Ma NPs were successfully taken up by RAW264.7 cells and achieved effective lysosomal escape after internalization.

### 2.3 In Vitro Anti‐Inflammatory Ability and Polarization Regulation of a127/mito@ZIF@Ma NPs on Macrophages

It has been shown that miRNA‐127 plays a crucial role in regulating macrophage polarization, facilitating the transition from the M2 anti‐inflammatory to M1 pro‐inflammatory phenotype.^[^ ^33^, ^34^ ^]^ In this work, anti‐miRNA‐127 was delivered to inflammatory macrophage, with the objective of silencing miRNA‐127 and promoting the polarization of macrophage from M1 to M2, thereby mitigating the inflammatory response. First, we detected the silencing efficacy of miRNA‐127 by real‐time quantitative PCR (qRT‐PCR). The result showed that miRNA‐127 expression level was relatively low in normal RAW264.7 cells, but significantly increased following stimulation with LPS. Notably, treatment with a127@ZIF@Ma NPs resulted in a marked reduction in miRNA‐127 expression (Figure S5, Supporting Information).

**Figure 4:**
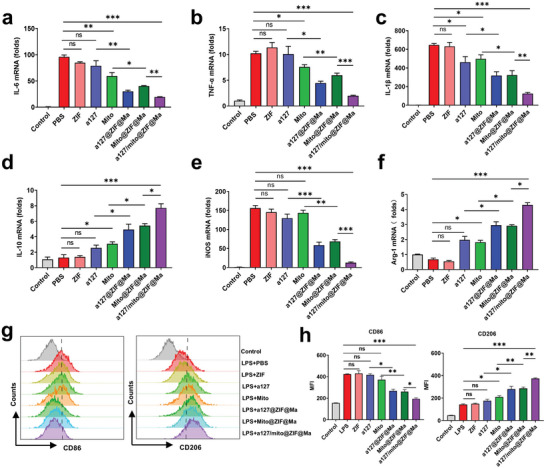
a127/mito@ZIF@Ma NPs inhibit the expression of pro‐inflammatory cytokines and facilitate the polarization of M1 macrophages toward M2 phenotype. The RAW264.7 cells were treated with various formulations, including ZIF, a127, Mito, a127@ZIF@Ma NPs, Mito@ZIF@Ma NPs, and a127/mito@ZIF@Ma. Cells were exposed to LPS (100 ng mL^−1^) for an additional 12 h apart from the control group. (a–d) The mRNA expression levels of IL‐6, IL‐1β, TNF‐α, and IL‐10 were measured by qRT‐PCR. (e,f) The mRNA expression levels of iNOS and Arg‐1 were measured by qRT‐PCR. (g,h) Representative flow cytometry histogram and average relative mean fluorescence intensity (MFI) for CD86 (left) and CD206 (right) on RAW264.7 cells. All the data are expressed as the mean ± SD (*n* = 3); ns, no significance; *p < 0.05, **p < 0.01, ***p < 0.001.

In order to detected the anti‐inflammatory ability and polarization regulation of a127/mito@ZIF@Ma NPs on macrophages, the expression of inflammation‐related factors was evaluated via qRT‐PCR after treatment with a127/mito@ZIF@Ma NPs. The results revealed a significant down‐regulation of pro‐inflammatory cytokines IL‐6, IL‐1β, and TNF‐α, along with an up‐regulation of the anti‐inflammatory cytokine IL‐10 (**Figure** **4**a–d). Additionally, the M1 macrophage marker iNOS was significantly down‐regulated, while the M2 macrophage marker Arg‐1 exhibited a significantly increase (Figure 4e,f). Flow cytometry analysis further confirmed these findings, showing that treatment with a127/mito@ZIF@Ma lead to a decrease in the expression level of CD86 (an M1 marker) and an increase in the expression level of CD206 (an M2 marker) in LPS‐stimulated RAW264.7 cells (Figure 4g,h). In summary, these results indicate that LPS‐stimulated macrophages can be effectively reprogrammed from pro‐inflammatory to anti‐inflammatory phenotype.

### 2.4 a127/mito@ZIF@Ma NPs Regulate Mitochondrial Function and Oxidative Phosphorylation In Vitro

**Figure 5:**
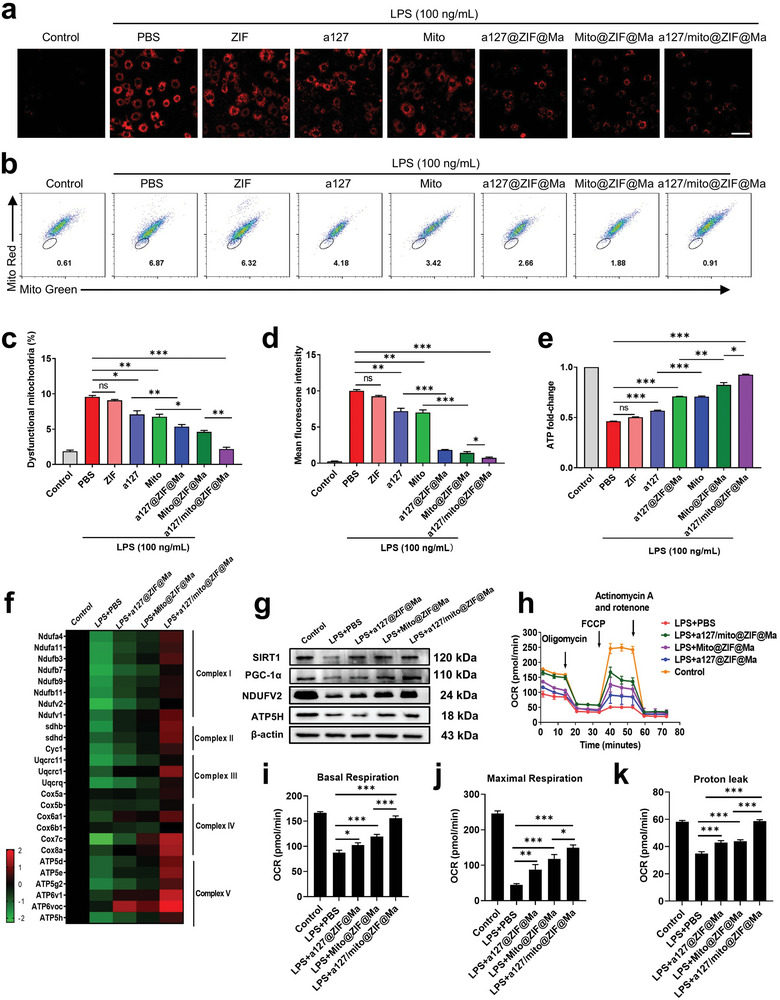
a127/mito@ZIF@Ma NPs improve macrophage mitochondrial function stimulated by LPS. RAW264.7 cells were pretreated with various formulations, including PBS, ZIF, a127, a127@ZIF@Ma, Mito, Mito@ZIF@Ma, and a127/mito@ZIF@Ma NPs for 12 h and treated with LPS (100 ng mL^−1^) for 12 h later. (a) MitoSOX‐Red staining of RAW264.7 cells. (b) Flow cytometry analysis of mitochondria staining using Mito Tracker Red and Mito Tracker Green. (c) Quantification of dysfunctional mitochondria staining with Mito Tracker Red and Mito Tracker Green. (d) Quantification of MitoSOX‐Red staining. (e) Measurement of mitochondrial ATP production in RAW264.7 cells. (f) Heatmap showing the expression of genes associated with mitochondrial respiratory chain complex‐related genes in MH‐S cells, as determined by qPCR with primer sequences provided in Table S1 (Supporting Information). (g) Western blot analyzed mitochondria‐related proteins. (h‐k) Measurement of oxygen consume rates (OCRs) using the Seahorse XFe96 Analyzer, from which baseline respiratory capacity, proton leakage, and maximal respiratory capacity were calculated. All the data are expressed as the mean ± SD (*n* = 3); ns, no significance; *p < 0.05, **p < 0.01, ***p < 0.001.

In order to evaluate the effect of a127/mito@ZIF@Ma NPs on mitochondrial function, RAW264.7 cells were subjected to various treatments, followed by stimulation with LPS. The results revealed that a significant increase in mitochondrial reactive oxygen species (ROS) within LPS‐induced macrophage inflammation model. As shown in **Figure** **5**a,d, various treatments resulted in a reduction of LPS‐induced mitochondrial ROS levels, with a particularly pronounced effect in lowering ROS levels in the a127/mito@ZIF@Ma group when compared to other experimental groups. Additionally, we used mitotracker‐Red and mitotracker‐Green to assess mitochondrial function alterations during LPS‐induced inflammation in macrophages. The results showed a marked decrease in mitochondrial activity in the LPS‐treated group (Figure 5b,c). However, consistent with prior observation, mitochondrial function was significantly restored following treatment with a127/mito@ZIF@Ma NPs (Figure 5b,c). Furthermore, the adenosine triphosphate (ATP) levels in RAW264.7 cells were examined. The results showed that a significant reduction of ATP level in the LPS‐treated group. However, treatment with a127@ZIF@Ma NPs and Mito@ZIF@Ma NPs resulted in a notable enhancement of ATP levels when compared to the administration of free anti‐miRNA‐127 and free mitochondria. Notably, the increase in ATP levels was particularly pronounced in cells treated with the a127/mito@ZIF@Ma NPs (Figure 5e). Next, we analyzed the mRNA expression of genes associated with mitochondrial respiratory chain‐related genes using qPCR. The results showed that both a127@ZIF@Ma NPs and Mito@ZIF@Ma NPs enhanced the expression of these genes, with a127/mito @ZIF@Ma NPs exhibiting the most pronounced efficacy (Figure 5f). Furthermore, the expression of mitochondria‐related proteins was evaluated, which was consistent with previous results, as these proteins were significantly up‐regulated following treatment with a127/mito@ZIF@Ma NPs (Figure 5g).

To further investigate the respiratory capacity of RAW264.7 cells post‐treatment, a seahorse analyzer was utilized. The results showed a significantly reduction in respiratory capacity of RAW264.7 cells following LPS treatment compared to the control group. However, treatment with a127/mito@ZIF@Ma NPs resulted in a notable improvement in mitochondria respiratory capacity, which was the most obvious among these treatment groups (Figure 5h–k). In summary, a127/mito@ZIF@Ma NPs can significantly improve the mitochondrial‐related functions in LPS‐induced RAW264.7 inflammation models.

### 2.5 a127/mito@ZIF@Ma NPs are Mainly Uptake by Macrophages In Vivo

**Figure 6:**
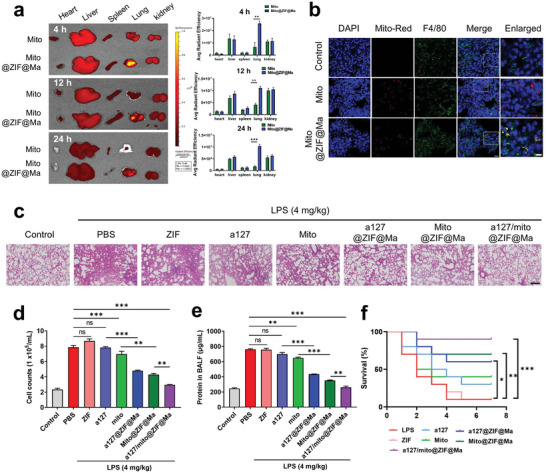
Administration of a127/mito@ZIF@Ma NPs alleviate endotoxin‐induced lung injury. (a) Living imaging of vital organs was conducted at 4 or 24 h after administration of a127/mito@ZIF@Ma NPs, with mitochondria being labeled using Mito‐tracker Red. (b) The colocalization of F4/80 (green) with internalized mitochondria (red) in lung tissues. Scale bar, 10 µm. (c) Representative H&E staining of lung tissues. Scale bar, 200 µm. (d) BALF cell counts; (e) Total protein level. (f) Survival rate of mice challenged with a high dose of LPS (10 mg kg^−1^) and then treated with a127/mito@ZIF@Ma NPs, as well as other formulations. Kaplan–Meier survival plots were generated with *n* = 10 mice per group. All the data are expressed as the mean ± SD (*n* = 3); ns, no significance; *p < 0.05, **p < 0.01, ***p < 0.001.

In order to explore the in vivo targeting ability of a127/mito@ZIF@Ma toward macrophages, mitotracker‐Red was used to label mitochondria and Mito@ZIF@Ma NPs, respectively. LPS (4 mg kg^−1^) was used to induce an acute lung injury model in mice through airway instillation. Subsequently, LPS‐treated mice received tail vein injections of either mitochondria or a127/mito@ZIF@Ma NPs. After injection for 4, 12, or 24 h, mice were sacrificed, and the distribution of the administered drugs across various organs were examined using a living imaging technology (**Figure** **6**a). The results showed a significant accumulation of fluorescence in the lungs, with prolonged retention observed. Notably, a127/mito@ZIF@Ma NPs exhibited an obvious increase in lung accumulation compared to the free mitochondria‐treated group at all time points (Figure 6a). Additionally, the targeting ability of a127/mito@ZIF@Ma toward lung macrophages was evaluated. Lung macrophages were identified using anti‐F4/80 antibodies, while mitochondria were labeled with mitotracker Red. In contrast to the group exposed to free mitochondria, a127/mito@ZIF@Ma NPs exhibited a significant co‐localization with lung macrophages. These results suggested that a127/mito@ZIF@Ma NPs possess the ability to remain in the lungs for extended periods and specifically target lung macrophages, particularly in the LPS‐induced acute lung injury model (Figure 6b).

### 2.6 a127/mito@ZIF@Ma NPs Alleviated LPS‐Induced Acute Lung Injury

Considering the powerful capacity of a127/mito@ZIF@Ma NPs to restore inflammatory macrophages in vitro and their accumulation within pulmonary tissue, the in vivo efficacy of these nanoparticles was further explored in lung diseases. ALI mice model was established through an endotracheal instillation of LPS, followed by administration of various treatment including PBS, ZIF, a127, mito, a127@ZIF@Ma, Mito@ZIF@Ma, and a127/mito@ZIF@Ma NPs. Histological examination showed thickening of the alveolar walls, structural disturbance of the lung architecture, and heightened cellular infiltration following LPS treatment. Conversely, a decrease in alveolar wall thickness was observed post‐treatment, particularly with a127/mito@ZIF@Ma NPs, which markedly alleviated pulmonary immune pathology and diminished the infiltration of inflammatory cells (Figure 6c). To evaluate the effects of these treatments, BLAF was collected from each group after 24 h of administration and centrifuged to analyze the protein content in the supernatant, and the cell counts in the BLAF were also recorded. The results showed a significant increase in both protein content and cell numbers in LPS‐treated mice with acute lung injury, whereas administration of a127/mito@ZIF@Ma NPs resulted in a reduction of protein penetration and total cell counts and a significant extension of survival time (Figure 6d–f).

**Figure 7:**
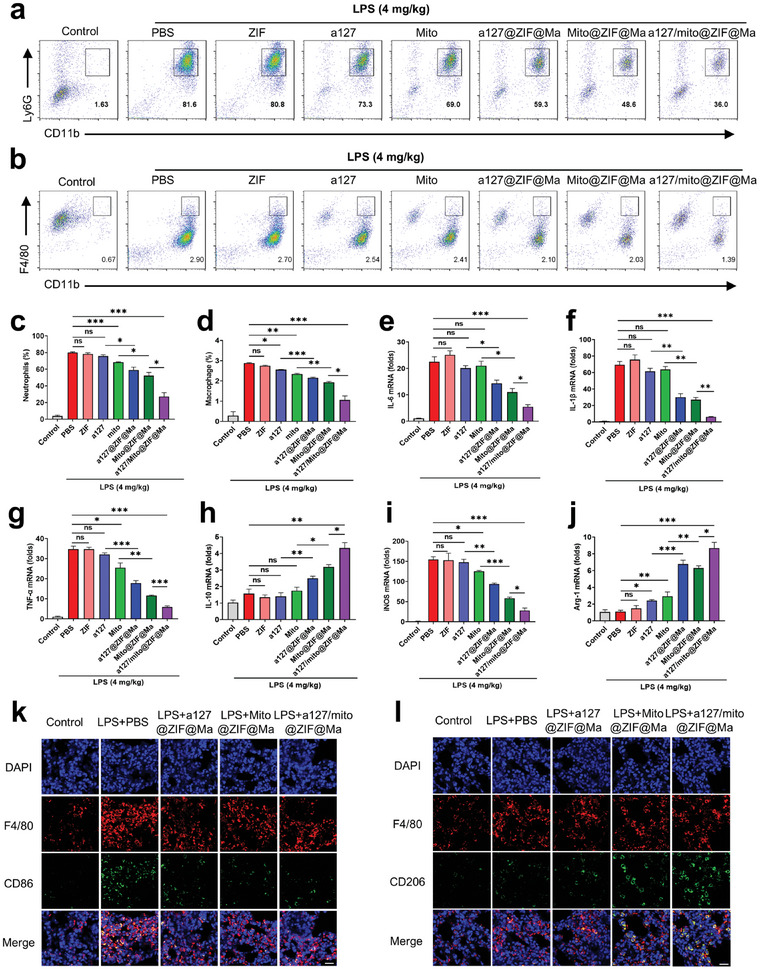
a127/mito@ZIF@Ma NPs attenuate acute lung injury by inhibiting inflammatory responses and modulating macrophage polarization. C57BL/6 mice were treated with LPS (4 mg kg^−1^, i.t.), followed by the administration of a127/mito@ZIF@Ma NPs (*n* = 3). After 24 h, mice were sacrificed and subjected to functional analysis. (a,b) Flow cytometry analysis of CD45^+^CD11b^+^F4/80^+^ macrophages and CD45^+^CD11b^+^Ly6G^+^ neutrophils in BALF. (c,d) Quantification of neutrophils and macrophages in BALF. (e–j) The expression levels of cytokines in lung tissues using qPCR. (k,l) Immunofluorescence and immunohistochemical staining of M1 and M2 macrophages through distinct markers. Scale bar, 20 µm. All the data are expressed as the mean ± SD; ns, no significance; *p < 0.05, **p < 0.01, ***p < 0.001.

Endotracheal instillation of LPS consistently resulted in an increased percentage of CD45^+^Ly6G^+^CD11b^+^ neutrophils and CD45^+^F4/80^+^CD11b^+^ macrophages, which are two predominant pro‐inflammatory cell populations in acute lung injury. As shown in **Figure** **7**a–d, treatment with a127/mito@ZIF@Ma NPs significantly mitigated the infiltration of macrophages and neutrophils in the lung tissue. Therefore, these data suggested that a127/mito@ZIF@Ma NPs exert a regulatory influence on lung immune cell populations. Furthermore, a127/mito@ZIF@Ma treatment led to a significant decrease in the expression of pro‐inflammatory cytokines, including IL‐6, TNF‐α, and IL‐1β, while enhancing the expression of anti‐inflammatory cytokine IL‐10 (Figure 7e–h). Additionally, the mRNA expression levels of M1 macrophage marker (iNOS) and M2 macrophage marker (Arg‐1) in lung tissue were examined (Figure 7i,j). qPCR results showed a significant upregulation of iNOS expression following the administration of LPS. In contrast, a127/mito@ZIF@Ma treatment resulted in a notable downregulation of iNOS expression and upregulation of Arg‐1 expression. This finding suggested that a127/mito@ZIF@Ma NPs may exert an anti‐inflammatory effect by promoting the polarization of macrophage from M1 to M2 phenotype. Next, immunofluorescence was used to further investigate the polarization process from M1 to M2 macrophages. Compared to the control group, a127/mito@ZIF@Ma treatment lead to a significant reduction in the M1 marker CD86 and an increase in M2 marker CD206 (Figure 7k,l), thereby demonstrating that a127/mito@ZIF@Ma NPs can effectively alter macrophage phenotypes from M1 to M2.

### 2.7 a127/mito@ZIF@Ma Protects Mice from Influenza Pneumonia and Confers Survival Benefits

**Figure 8:**
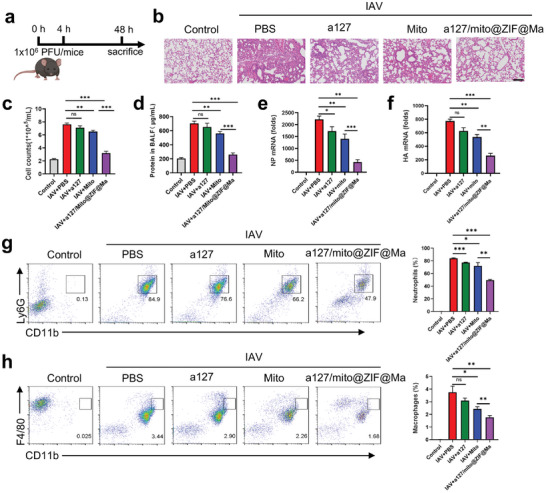
The therapeutic efficacy of a127/mito@ZIF@Ma NPs on influenza pneumonia. (a) The schematic design and treatments for influenza pneumonia (*n* = 3) (Created by Biorender). (b) H&E staining of lung tissues. Scale bar, 200 µm. (c) BALF cell counts. (d) Total protein level. (e,f) viral hemagglutinin (HA) and nuclear protein (NP) mRNA expression levels in lungs measured by qPCR. (g,h) Flow cytometry analysis of CD45^+^CD11b^+^Ly6G^+^ neutrophils and CD45^+^CD11b^+^F4/80^+^ macrophages in BALF. All the data are expressed as the mean ± SD; ns, no significance; *p < 0.05, **p < 0.01, ***p < 0.001.

Influenza virus has become a serious threat to global health, with its incidence often associated with the disruption of lung homeostasis and the hyper‐inflammatory responses. In this study, we also investigated the potential therapeutic effects of a127/mito@ZIF@Ma on pneumonia induced by H1N1 virus. To establish lung infection model, we instilled the H1N1 virus (1 × 10^6^ PFU) into the mice airways, followed by the administration of PBS, a127, mito, and a127/mito@ZIF@Ma NPs to evaluate the therapeutic outcomes (**Figure** **8**a). As shown in Figure 8b,e,f, the therapeutic efficacy was associated with reduced levels of viral hemagglutinin (HA) and nuclear protein (NP). The results showed a substantial reduction in protein leakage in BALF and a significant decrease in viral load following treatment with a127/mito@ZIF@Ma NPs (Figure 8c,d). In addition, the administration of a127/mito@ZIF@Ma NPs resulted in a notable improvement in lung pathology and a reduction of immune cell infiltration in mice infected with influenza A virus (IAV) (Figure 8b–f). qPCR and flow cytometric analysis revealed that a127/mito@ZIF@Ma NPs treatments lead to a decrease in the expression of pro‐inflammatory cytokines, as well as a reduction in the infiltration of neutrophils (Figure 8g; Figure S6a–d, Supporting Information). Additionally, the expression of F4/80, a marker associated with macrophage, was significantly up‐regulated after IAV induction (Figure 8h); However, this expression was markedly down‐regulated after treated with a127/mito@ZIF@Ma NPs, concomitantly with a significant increase in M2 macrophages (Figure 8h; Figure S6e,f, Supporting Information). Taken together, these findings suggested that a127/mito@ZIF@Ma NPs treatment protected mice from influenza pneumonia by restoring homeostasis with the lung and preserving tissue integrity.

### 2.8 Safety Profile of a127/mito@ZIF@Ma Therapies

To evaluate the in vivo biosafety of a127/mito@ZIF@Ma NPs therapies, a histological examination of the main organs in mice across various treatment groups was conducted. H&E staining revealed no significant abnormalities in the heart, liver, spleen, and kidney tissues, thereby reinforcing the biocompatibility of the treatment (Figure S7, Supporting Information). Additionally, a clinical serum routine analysis of all experimental groups was carried out, which including measurements of white blood cells (WBC), hemoglobin (HGB), platelets (PLT). The serum routine analysis demonstrated negligible differences when compared to PBS group (Figure S8a–c, Supporting Information). Furthermore, a biochemical analysis of blood parameters indicated that the levels of alanine aminotransferase (ALT), aspartate aminotransferase (AST), blood urea nitrogen (BUN), remained within normal ranges, indicating that the treatment did not adversely affect liver and kidney function (Figure S8d–f, Supporting Information). In addition, hemolytic tests of ZIF‐8, a127, Mito, a127@ZIF@Ma, Mito@ZIF@Ma, and a127/mito@ZIF@Ma NPs at designated concentrations were performed to further evaluate hemocompatibility. In comparison to the positive control group, the supernatant in all treated groups exhibited a clear coloration (Figure S9, Supporting Information), suggesting that there was no lysis of red blood cells, which aligns with the findings from the quantitative analysis. In summary, these preliminary findings indicated that a127/mito@ZIF@Ma NPs did not cause obvious side effects during treatment, showing their potential as a safe therapeutic candidate for acute lung injury conditions.

## 3 Discussion

Mitochondrial transplantation has emerged as a promising therapeutic approach for the treatment of diseases associated with mitochondrial dysfunction. Comparing with other techniques that involve editing the mitochondrial genome, the direct delivery of mitochondria offers a more straightforward means of replacing damaged mitochondria to restore cellular function.^[^ ^12^, ^35^, ^36^, ^37^ ^]^ Previous research has indicated that exogenous mitochondria can be transferred to recipient cells through phagocytosis.^[^ ^38^ ^]^ However, the efficiency of this transfer is significantly affected by the quantity of extracellular mitochondria present during co‐culture with the recipient cells, as the uptake of exogenous mitochondria by these cells occurs randomly and lacks targeting capacity. Furthermore, In vivo studies have revealed that that a substantial proportion of transplanted mitochondria remains in the interstitial fluid between cells, with only a limited number being internalized into the cells. Therefore, there is a pressing need for more effective strategies to deliver mitochondria within complex physiological environments to specific organs and cells. In this study, we constructed a biomimetic MOF nanocarrier to deliver healthy mitochondria. MOF nanoparticles was conjugated with mitochondria by a bio‐orthogonal chemistry strategy, and showing a significant delivery efficiency both in vitro and in vivo. The introduction of exogenous mitochondria into inflammatory macrophage cells has shown the mitigation of reactive oxygen species production, the enhanced ATP synthesis, the restored membrane potential, and the improved mitochondrial respiration in the acute lung injure models, thereby facilitating the recovery of cellular energy metabolism function.

Exogenous mitochondrial transplantation is the mainstay of mitochondrial therapy. Clinical sources of exogenous mitochondria are mainly extracted from the patient's own body, such as skeletal muscle and rectus abdominis muscle.^[^ ^39^, ^40^ ^]^ Autologous mitochondrial transplantation is safe with no patients experiencing complications after mitochondrial injections, and their blood parameters are similar to those patients who did not receive mitochondrial injections.^[^ ^41^ ^]^ In addition, the antigenicity of allogenic mitochondrial is weak and allows for tolerance induction.^[^ ^42^ ^]^ Therefore, mitochondrial transfer will provide promising therapeutic opportunities for mitochondrial medicine.

As pivotal effector cells of innate immunity, macrophage play a crucial role in the recognition and elimination of invasive pathogens, thereby contributing to the maintenance of immune system homeostasis.^[^ ^43^, ^44^ ^]^ Macrophage have remarkable plasticity, polarizing toward a pro‐inflammatory M1 phenotype in response to inflammatory stimuli and transitioning to an anti‐inflammatory M2 phenotype during tissue repair.^[^ ^45^, ^46^ ^]^ MicroRNAs (miRNAs) play a crucial role in the regulation of gene expression associated with macrophage polarization and macrophage inflammatory response.^[^ ^47^, ^48^, ^49^, ^50^ ^]^ While miRNAs can be internalized by macrophages through endocytosis, their mimics typically exhibit a deficiency in lysosomal escape, resulting in degradation and a subsequent reduction in their functional efficacy. Therefore, the development of effective interventions aimed at promoting macrophage polarization is vital for attenuating the pathological processes associated with acute lung injury.

In our investigation, we employed a synergistic delivery strategy that significantly diminished the inflammation within lung tissue. This result was attributed to the targeting ability of the ZIF‐8‐encapsulated macrophage membranes, which enhanced phagocytosis and subsequent internalization. Furthermore, the pH‐responsive properties of ZIF‐8 facilitated the endosomal escape and facilitate the release of anti‐miRNA‐127 and mitochondria. Ultimately, this synergistic delivery strategy yielded anti‐inflammatory effects and improved cellular energy supply, thereby offering an innovative therapeutic strategy for the treatment of pathogen‐ or virus‐induced pneumonia.

## 4 Conclusion

In summary, we have fabricated a biomimetic MOF nanocarrier with macrophage‐targeting capability and validated their utility in the treatment of ALI. Macrophage cell membrane‐coated MOF nanoparticles not only effectively enhanced the delivery efficiency of mitochondria and anti‐miR‐127 into inflammatory macrophages, but also facilitating their escape from endosome and lysosome. Upon entering inflammatory macrophage cells, anti‐miR‐127 effectively silenced miRNA‐127, promoting the polarization of macrophages from M1 to M2 phenotype, which in turn reduces the inflammatory response. In addition, the transfer of exogenous mitochondria restored the mitochondrial functions, including ATP production, mitochondrial membrane potential, levels of reactive oxygen species, and the oxygen consumption rate of the target cells. Furthermore, in vivo experiments indicated that the administration of a127/mito@ZIF@Ma nanoparticles significantly reduced lung inflammation in ALI models induced by both bacterial and virus pathogens, which offers a novel therapeutic approach for the clinical management of acute lung injury.

## 5 Experimental Section

### Materials

Zn(NO_3_)_2_·6H_2_O, 2‐methylimidazole (2‐MIM), and lipopolysaccharide (LPS; O55:B5) were purchased from Sigma–Aldrich (Steinheim, Germany). Anti‐miRNA‐127 (5′‐AGCCAAGCUCAGACGGAUCCGA‐3′) and FAM‐labeled anti‐miR‐127 oligonucleotides were obtained from Genepharma (Shanghai, China). Me‐Tetrazine‐DBCO (TZ‐DBCO), (4E)‐TCO‐NHS ester (TCO‐NHS), and Ac4ManNAz were purchased from Confluore (Xian, China). Mitochondria isolation kit, MitoTracker Green, and Mito Tracker Red were purchased from Beyotime (Shanghai, China). Mito SOX was purchased from Invitrogen (Waltham, USA). Antibodies against β‐actin, F4/80, CD11b, Tom20, Sirt1, PGC‐1α, NDUFV2, and ATP5H were purchased from Cell Signaling (Beverly, USA).

### Cell Culture and Propagation of Influenza A Virus

RAW264.7 mouse macrophage cell line and Madin–Darby canine kidney (MDCK) cell line were obtained from the American Type Culture Collection (ATCC, USA) and cultured in complete DMEM medium according to the instructions. Influenza A virus (strain A/Puerto Rico/8/1934 H1N1) was propagated in MDCK cells. The culture medium was harvested upon the observation of a significant cytopathic effect. After titration using a plaque assay, media containing influenza A virus were aliquoted and stored at −80 °C for subsequent experiments.

### Extraction of Azido‐Labeled Macrophage Plasma Membrane

RAW264.7 were incubated in culture medium containing Ac4ManNAz (50 µm) for 3 days. Subsequently, culture medium was replaced with PBS containing Tz‐DBCO (50 µm) and incubated for 30 min. After washing with PBS, RAW264.7 were collected by centrifugation and resuspended in a hypotonic buffer. Cells were disrupted using Dounce homogenization, and plasma membrane was isolated from lysates through differential centrifugation (1000 g for 10 min, 10 000 g for 25 min, and 100 000 g for 1 h). BCA protein assay was performed to quantify the amount of protein, which was correlated with the amount of plasma membrane.

### Mitochondrial Isolation and Surface Modification

Fresh mitochondria were isolated from RAW264.7 cells using a commercial mitochondria isolation kit. To introduce the TCO molecule on mitochondrial surface, mitochondria were incubated with TCO‐NHS (50 µm) for 30 min and unreacted TCO molecules were removed by centrifugation at 3500 g for 10 min. All procedures were conducted on ice to maintain the bioactivity of mitochondria.

### Preparation of a127@ZIF@Ma Nanoparticles

a127@ZIF nanoparticles were prepared via a one‐pot synthesis strategy as follows: 0.5 mL Zn(NO_3_)_2_·6H_2_O (30 mg mL^−1^) and 40 µL anti‐miR‐127 (100 µm) were first mixed and stirred at 30 °C for 5 min. 1 mL 2‐methylimidazole dissolved in methanol (33 mg mL^−1^) was slowly added into above solution and the mixture was stirred for another 5 min. a127@ZIF nanoparticles were collected by centrifugation and washed three times with ethanol. Furthermore, a127@ZIF nanoparticles were lyophilized and weighed to evaluate the synthesis efficiency.

To prepare membrane‐coated a127@ZIF (a127@ZIF@Ma) nanoparticles, plasma membrane was suspended with PBS at 2 mg mL^−1^ and extruded successively through 1000, 400, and 200 nm polycarbonate porous membranes by an Avanti mini extruder to prepare the membrane vesicles. a127@ZIF (1 mg mL^−1^) was mixed with macrophage‐derived membrane vesicles, sonicated in a sonicator bath for 3 min, and repeatedly extruded through 200 nm polycarbonate porous membranes for 30 times. a127@ZIF@Ma nanoparticles were collected by centrifugation at 12 000 rpm for 10 min.

### Construction of Engineered Mitochondrial Conjugated with a127@ZIF@Ma

a127@ZIF@Ma were conjugated to mitochondria through a bioorthogonal chemistry approach as previously reported. Briefly, TCO‐labeled mitochondria (2.5–50 µg, µg of protein) and DBCO‐labeled a127@ZIF@Ma (15 µg mL^−1^) were mixed together and incubated in mitochondrial storage solution for 30 min. Engineered mitochondria were further centrifuged at 3500 g for 10 min and washed twice with the storage solution to obtain the final products (a127/mito@ZIF@Ma). a127/mito@ZIF@Ma formulation was freshly prepared before experiment and used as soon as possible.

### Transmission Electron Microscopy

Mitochondria or a127/mito@ZIF@Ma was fixed with 2.5% glutaraldehyde (Solarbio, China). The fixed samples were dehydrated through a series of ethanol and embedded in epoxy resin. Ultrathin sections (70 nm) were prepared by using a diamond knife mounted on an Ultracut (Leica, Japan). The sections were placed on copper grids, counter‐stained with 2% uranyl acetate, and photographed with transmission electron microscope.

### Mito Tracker Green and Red Staining

In accordance with the instructions of the mitochondrial indicators Mito Tracker Red and Mito Tracker Green (Beyotime, China), RAW264.7 cells were incubated with 100 nm Mito Tracker Green for 30 min at 37 °C to measure the mitochondrial content, or with 0.5 µm Mito Tracker Red for 30 min at 37 °C to detect the mitochondrial membrane potential. Subsequently, the cells were washed with warmed staining buffer and analyzed by flow cytometry (BD Biosciences).

### JC‐1 Staining

Mitochondria was stained with mitochondrial membrane potential probe JC‐1 (Beyotime, China) according to the manufacturer's instructions. Fluorescent images of mitochondria, which labeled with the JC‐1 probe, were obtained utilizing a confocal microscope.

### Encapsulation Efficiency and Controlled Release of anti‐miRNA‐127

To determine the encapsulation efficiency, FAM‐labeled anti‐miRNA‐127 was dissolved in different concentrations and the fluorescence intensity was measured. A calibration curve (Y = 40.865X+3393.1) of fluorescence intensity versus the concentration of FAM‐labeled anti‐miR‐127 was obtained.

After the synthesis of a127@ZIF NPs, the fluorescence intensity of FAM was detected after centrifugation and 5‐fold dilution, and the content of anti‐miRA‐127 was calculated to be 237.25 nm according to the standard curve, since the initial dose of anti‐miRNA‐127 was 2.67 µm. In addition, the molecular weight of anti‐miRNA‐127 and the concentration of the a127@ZIF are known to be 6650 g mol^−1^ and 0.6 g L^−1^, respectively.

Encapsulation Efficiency was defined as the ratio of the amount of drug contained within the nanoparticle to the total amount of drug added in the formulation of the nanoparticles. Drug loading capacity (DLC) was defined as the weight ratio of drug to nanoparticles. Accordingly, the encapsulation efficiency and drug loading capacity (DLC) of anti‐miR‐127 could be determined as follows. Encapsulation efficiency = (C _total anti‐miRNA‐127 –_ C _encapsulated anti‐miRNA‐127_)/ C _total anti‐miRNA‐127_ × 100% = (2760–237.25 nm)/ 2760 nm × 100% = 91.40%. Drug loading capacity (DLC) = M_anti‐miRNA‐127_/M_a127@ZIF_ × 100% = 2.43 µm × 6650 g mol^−1^ × 1.5 mL / 0.6 gL^−1^ × 1 mL = 4.0 wt.%

### In Vitro pH‐Responsive Behavior of a127@ZIF Nanoparticles

a127@ZIF nanoparticles were incubated in the PBS solution with pH levels of 5.5 or 7.4, respectively. The supernatant was sampled at a predetermined time interval, and the quantity of released anti‐miRNA‐127 was subsequently assessed using fluorescence spectroscopy.

### Characterization of Mitochondria Transfer

RAW264.7 cells were resuspended in prewarmed (37 °C) MitoTracker staining solution and incubated for 30 min under appropriate growth conditions. After staining, cells were washed three times with phosphate‐buffered saline (PBS) and resuspended in fresh prewarmed medium. RAW264.7 cells were harvested according to the experiment and examined using fluorescence spectroscopy.

### In Vitro Cellular Uptake

In a typical experiment, RAW264.7 cells were counted and plated in a 35 mm confocal dish. Anti‐miRNA‐127, conjugated with FAM fluorophore, was synthesized and encapsulated within ZIF‐8 nanoparticles to enable the visualization of its localization. FAM‐a127@ZIF and FAM‐a127@ZIF@Ma were added into the culture medium and incubated with RAW264.7 cells for 12 h. To induce an inflammatory response in the RAW 264.7 cells, lipopolysaccharide (LPS, 100 ng mL^−1^) was simultaneously added into the culture medium. After incubation for an additional 12 h, RAW264.7 cells were fixed and the cellular uptake efficiency of a127@ZIF, a127@ZIF@Ma, Mito@ZIF@Ma, a127@ZIF@Ma, and a127/mito@ZIF@Ma was examined using a confocal microscope.

To evaluate the cellular uptake efficiency of mitochondria and Mito@ZIF@Ma, RAW264.7 cells were prelabelled with Mito‐tracker prior to being plated in a confocal dish. Freshly isolated mitochondria (15 µg mL^−1^) and synthesized Mito@ZIF@Ma (15 µg mL^−1^) were incubated with RAW264.7 for 12 h. LPS were used in a way as mentioned above.

To monitor the release process of anti‐miRNA127 and mitochondria into the cytoplasm, isolated mitochondria were labeled with Mitotracker Red and conjugated with FAM‐a127/mito@ZIF@Ma nanoparticles. RAW246.7 cells were incubated with a127/mito@ZIF@Ma (15 µg mL^−1^) for 12 h, and imaging was conducted at predetermined time points.

### Quantitative Real‐Time PCR (qRT‐PCR)

Total RNA was extracted from RAW264.7 cells using Trizol regent (Thermo, USA) and transcribed accordance with the protocols in Reverse Transcription Kit (Yeasen, Shanghai, China). The resulting complementary DNA (cDNA) samples were diluted and quantified via real‐time PCR employing SYBR Green Master Mix (Yeasen, Shanghai, China). Gene expression levels were assessed using the 2^−ΔΔCt^ method and normalized with β‐actin. The primer sequences were detailed in Table S1 (Supporting Information).

### Immunoblot

Cell lysates were prepared by RIPA buffer containing protease inhibitor cocktail and phosphatase inhibitor cocktail (YEASEN). An equal amount of total protein was separated on SDS‐polyacrylamide mini‐gels and transferred onto PVDF membranes (Millipore). After being blocked in 5% skim milk (BD), the membranes were incubated with appropriate antibodies overnight, and then followed by the incubation of a secondary antibody conjugated with horseradish peroxidase. The immunoblotted proteins were visualized with an ECL detection reagent (Bio‐Rad).

### Intercellular ROS Measurement

RAW264.7 cells were washed three times with PBS and then treated with the medium supplemented with 5 µm Mito‐SOX staining solution (Invitrogen, USA). RAW264.7 cells were incubated in the dark for 20 min, and ROS levels were detected by a confocal microscope.

### Extracellular Flux Analysis

Oxygen consumption rates (OCR) were measured using an XFe96 Extracellular Flux Analyzer (Seahorse Bioscience, USA). Prior to the experiments, RAW264.7 cells were treated with a127@ZIF@Ma (30 µg mL^−1^), mito (15 µg mL^−1^, protein concentration), and a127/mito@ZIF@Ma (15 µg mL^−1^, protein concentration) for 12 h, followed by stimulated with LPS (100 ng mL^−1^) for another 12 h. The analysis was conducted under basal conditions or supplemented with the following reagents: oligomycin (1 µm), an ATP synthase inhibitor; FCCP (1 µm), a mitochondrial oxidative phosphorylation uncoupler; rotenone (100 nm), a mitochondrial complex I inhibitor, and antimycin A (1 µm), a mitochondrial complex III inhibitor. The basal oxygen consumption rate (OCR) was calculated by subtracting the OCR measured after treatment with rotenone and antimycin A from the OCR before oligomycin treatment. The maximal OCR was determined by subtracting the OCR after rotenone and antimycin A treatment from the OCR recorded after the addition of FCCP.

### ATP Measurement

RAW264.7 cell lysate was centrifuged at 12 000 rpm and 4 °C for 5 min and the supernatant was transferred into black 96‐well plates (100 µL per well). ATP level was assayed using an ATP quantification bioluminescent kit (Beyotime, China) according to the manufacturer's instructions.

### Biodistribution of a127/mito@ZIF@Ma

For the in vivo living imaging, a127/mito@ZIF@Ma was synthesized by coupling a127@ZIF@Ma (200 µg) with isolated mitochondria (100 µg, µg of protein) for a mouse with an approximate weight of 20 g. Prior to the experiment procedure, a127/mito@ZIF@Ma was prelabeled with Mito‐Tracker Red and subsequently administered to the mice via the tail vein injection. The biodistribution of a127/mito@ZIF@Ma was then assessed using the IVIS Lumina imaging system (Xenogen Corporation, USA).

### LPS and Influenza A Virus Induced Acute Lung Injury

All animal experiments were approved by the Ethics Committee of Zhejiang Shuren University (approval number: 230303), and all procedures related to animal experimentation according to the principles and guidelines established by the China Animal Protection Committee. C57BL/6 mice (male, 6–8 weeks) were used for the in vivo experiments.

The acute lung injury model was established in C57BL/6 mice by administering 50 µL of LPS (4 mg kg^−1^) or 50 µL of influenza A virus (Dose) through airway perfusion. Four hours later, ZIF@Ma, a127@ZIF@Ma, Mito@ZIF@Ma, and a127/mito@ZIF@Ma were administered via tail vein injection. The mice were monitored for 24 h before being euthanized for subsequent experiments.

### Isolation and Characterization of BALF Cells

Mice were sacrificed 24 h after drug administration, and alveolar lavage fluid was collected and centrifuged at 1500 rpm for 5 min. The total number of precipitated cells in the bronchoalveolar lavage fluid from each mouse was counted using a microscope, and the supernatant was collected for protein content determination. Subsequently, specific biomarkers for monocyte‐derived macrophages (anti‐CD45^+^, anti‐F4/80^+^, anti‐CD11b^+^) and neutrophils (anti‐CD45^+^, anti‐CD11b^+^, anti‐Ly6G^+^) were used to label the cell populations, which were then analyzed by flow cytometry.

### Immunofluorescence Staining

Lung tissue was systematically immersed in 20% and 30% sucrose solution for dehydration and sedimentation. The dehydrated tissue was then embedded in OTC and rapidly frozen for sectioning, with the sections cut to a thickness of 7 µm. The frozen slice was blocked with 3% BSA for 1 h and incubated overnight with primary antibody against F4/80 (1:200), CD86 (1:200), and CD206 (1:200). The slices were further stained with Alexa Fluor 594 goat anti‐rabbit or FITC goat anti‐mouse IgG (1:1000) for 1 h in the dark. After washing, the slices were stained with DAPI (1:1000) for 10 min and then imaged using confocal microscope.

### Hematoxylin and Eosin Staining

Lung tissues were washed thoroughly with PBS and then fixed in 4% PFA for 24 h. Paraffin‐embedded lung was sliced into 7 µm sections and stained with hematoxylin and eosin following standard procedure, then analyzed using an inverted microscope (DMi1, Leica).

### Statistical Analysis

Statistical analyses were performed using GraphPad Prism software. All the data are presented as mean ± SD derived from three independent experiments, employing Student's *t*‐test or one‐way analysis of variance (ANOVA) for comparisons. Survival curves were evaluated through Kaplan–Meier survival analysis and log‐rank test. *P* value < 0.05 was considered statistically significant.

## Conflict of Interest

The authors declare no conflict of interest.

## Author Contributions

X.S., C.J.C., and H.J.Y. contributed equally to this work. L.Y.S. and X.S. conceived the conceptualization and designed the experiment. C.J.C. carried out the experiments. X.S., H.J.Y., Z.Y.L., and L.Y.Z. analyzed data and wrote the paper. Q.J.L., L.J.L., B.Y.M., W.Z., and S.M.C. contributed to the scientific discussion of the article.

## Supporting information



Supporting Information

## Data Availability

The data that support the findings of this study are available in the supplementary material of this article.
