# Supplementary material for: Biomimetic MOF Nanocarrier‐Mediated Synergistic Delivery of Mitochondria and Anti‐Inflammatory miRNA to Alleviate Acute Lung Injury
Source: Adv Sci (Weinh). 2025 Feb 25;12(16):2416594. doi: 10.1002/advs.202416594 (PMC12021094; doi:10.1002/advs.202416594)
Supplement: Supplementary file 1 — Supporting Information [file ADVS-12-2416594-s001.docx]

Supporting Information

**Biomimetic MOF Nanocarrier-Mediated Synergistic Delivery of Mitochondria and Anti-Inflammatory miRNA to Alleviate Acute Lung Injury**

Xin Shou^*^, Changjiang Chen^*^, Hangjie Ying^*^, Zhiyun Liu, Lingyao Zeng, Qiujie Li, Lanjie Lei, Bingyong Mao, Wei Zhang, Shumao Cui^#^, Liyun Shi^#^

Xin Shou, Zhiyun Liu, Lingyao Zeng, Qiujie Li, Lanjie Lei, Liyun Shi

Key lab of Artificial Organs and Computational Medicine, Institute of Translational Medicine, Zhejiang Shuren University, Hangzhou, Zhejiang, 310015, China

Corresponding: Liyun Shi (Email: sly0202@zjsru.edu.cn)

Changjiang Chen, Wei Zhang

Department of Immunology, Nanjing University of Chinese Medicine, Nanjing, Jiangsu, 210023, China

Hangjie Ying

Department of Experiment Center, Zhejiang Cancer Hospital, Hangzhou Institute of Medicine (HIM), Chinese Academy of Sciences, Hangzhou, Zhejiang 310022, China

Bingyong Mao, Shumao Cui

State Key Laboratory of Food Science and Resources, Jiangnan University, Wuxi, Jiangsu 214122, China.

Corresponding: Shumao Cui (Email: cuishumao@jiangnan.edu.cn)


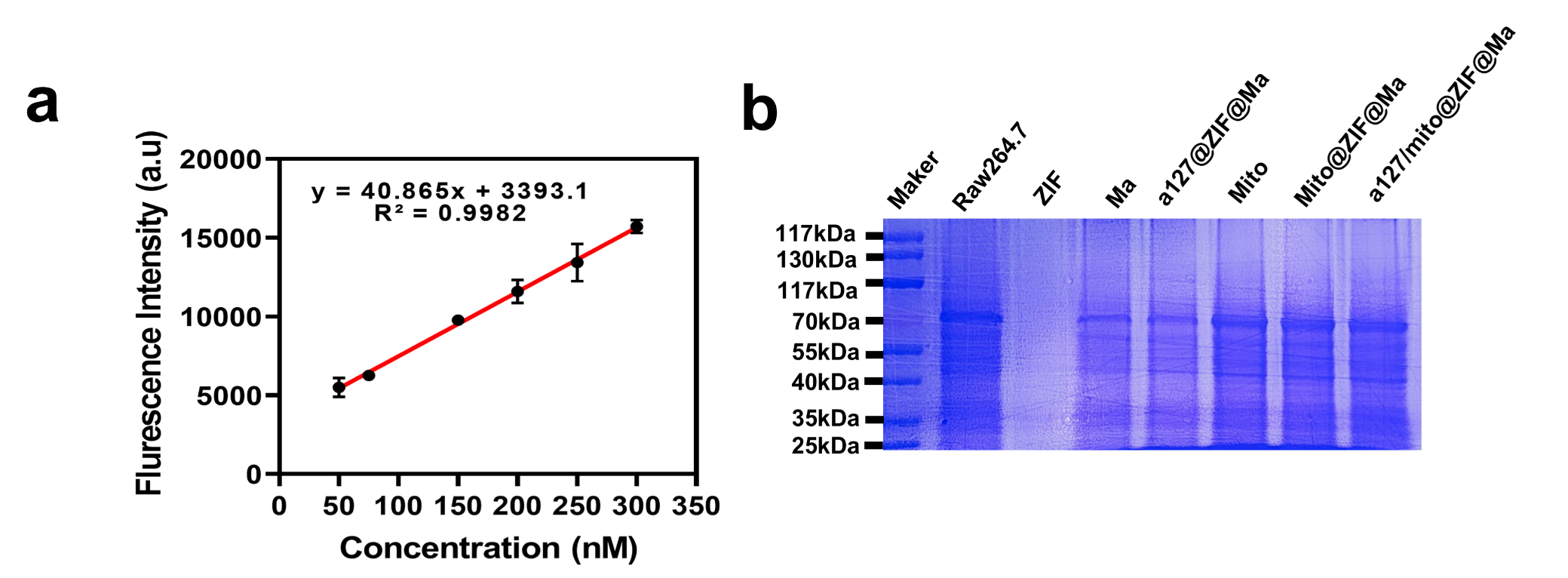


Figure S1. (a) Calibration curve of FAM fluorescence intensity with different concentrations of FAM-labeled anti-miR-127 for the drug loading capacity (DLC) calculation. Data were presented as mean ± SD (n = 3). (b)SDS-PAGE image of proteins in RAW264.7, ZIF-8, Ma, a127@ZIF@Ma, Mito, Mito@ZIF@Ma, a127/mito@ZIF@Ma NPs.


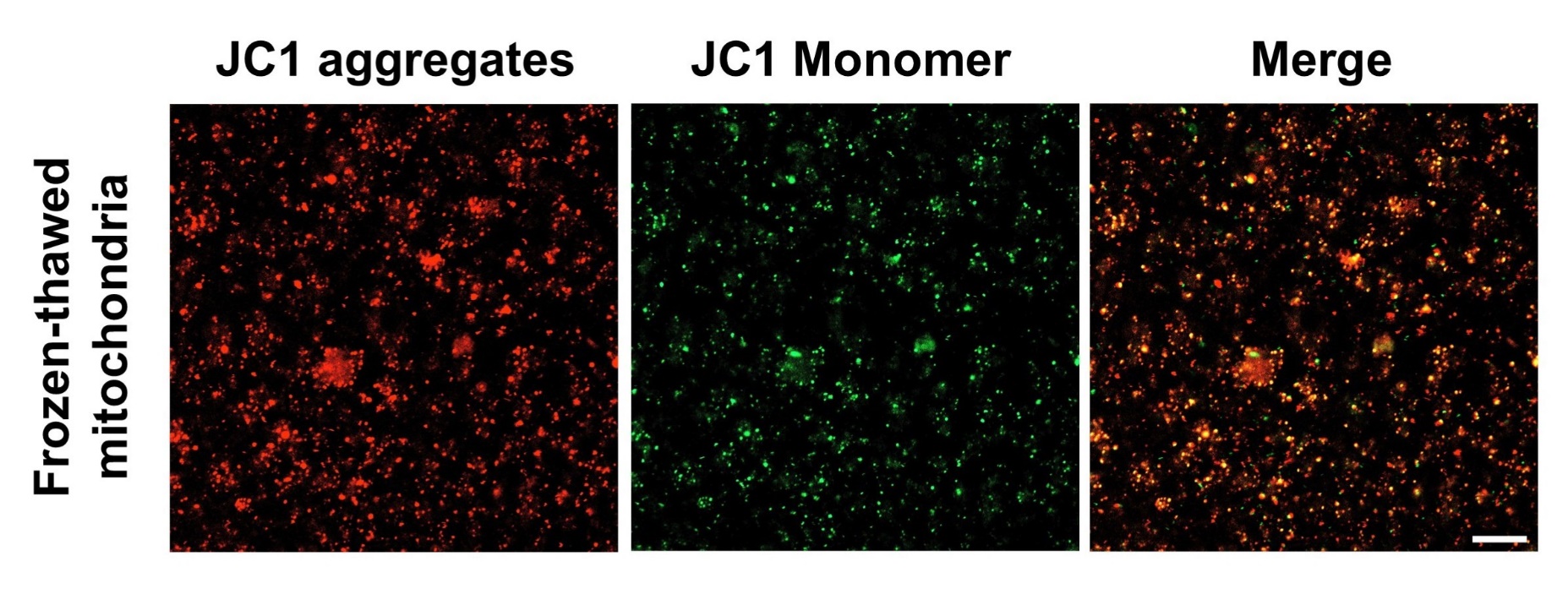


Figure S2. Representative images of JC1-stained mitochondria after frozen-thawed. Red: JC1 aggregates. Green: JC1 monomer. Scale bar, 20 μm.


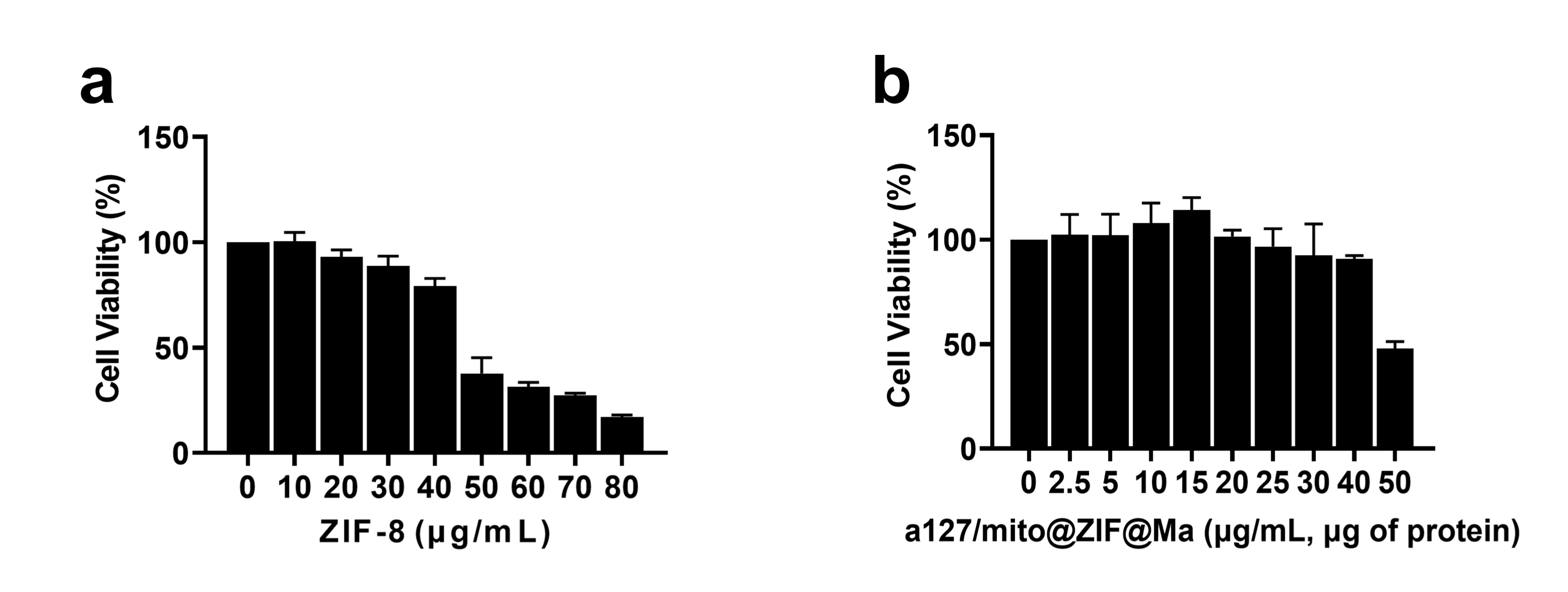


Figure S3. The cytotoxic effects of ZIF-8 (a) and a127/mito@ZIF@Ma (b) on RAW264.7 cells were measured after a 48-hour incubation with varying concentrations of nanoparticles. The experiments were repeated for three times (n = 3), and data were presented as mean ± SD.


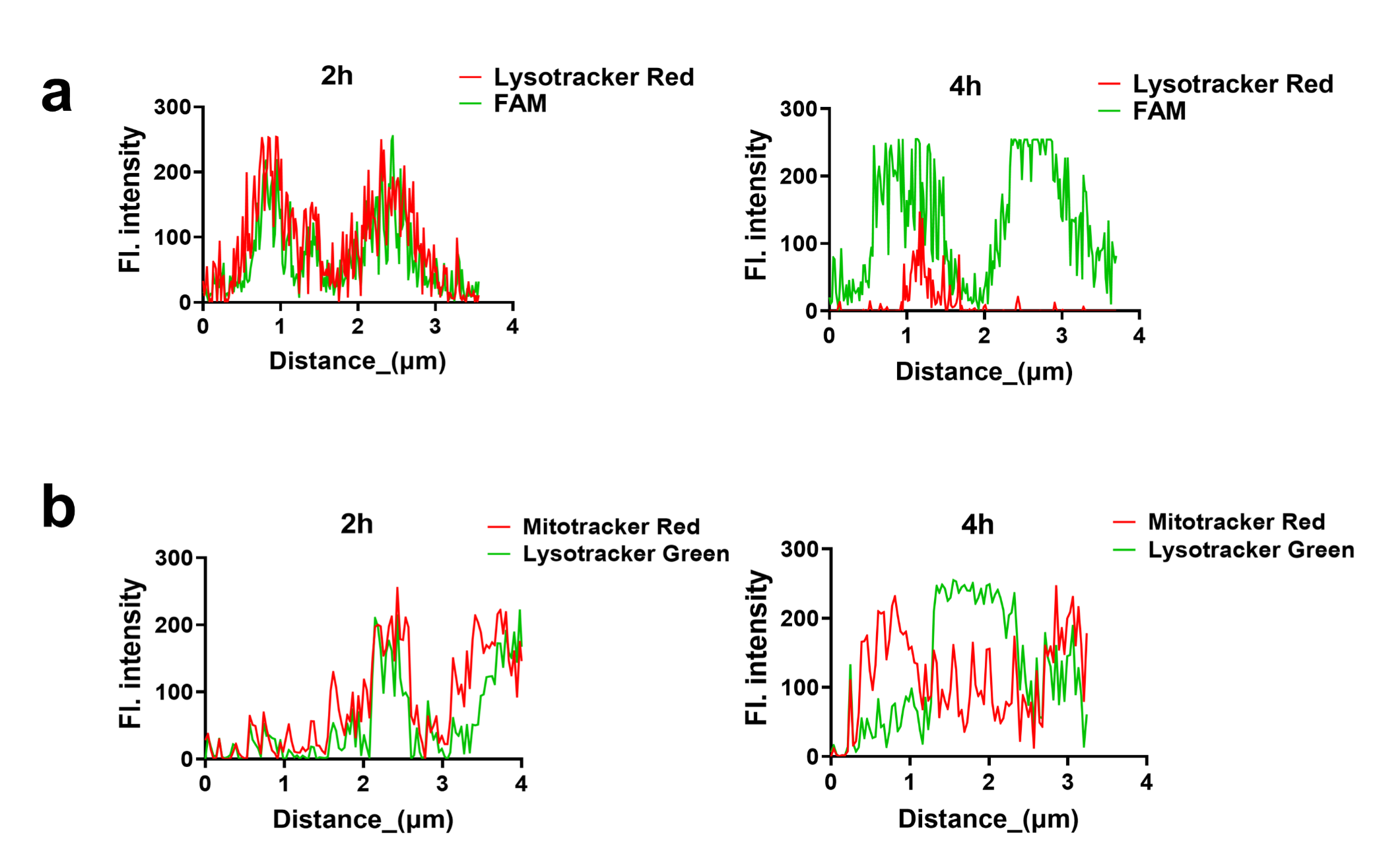


Figure S4. Intensity profiles of anti-miRNA-127 (a) and exogenous mitochondria (b) localized in RAW264.7 cells at 2 hour and 4 hours after incubation with a127/mito@ZIF@Ma NPs across the cell along the selected line (yellow line) in Figure 3.


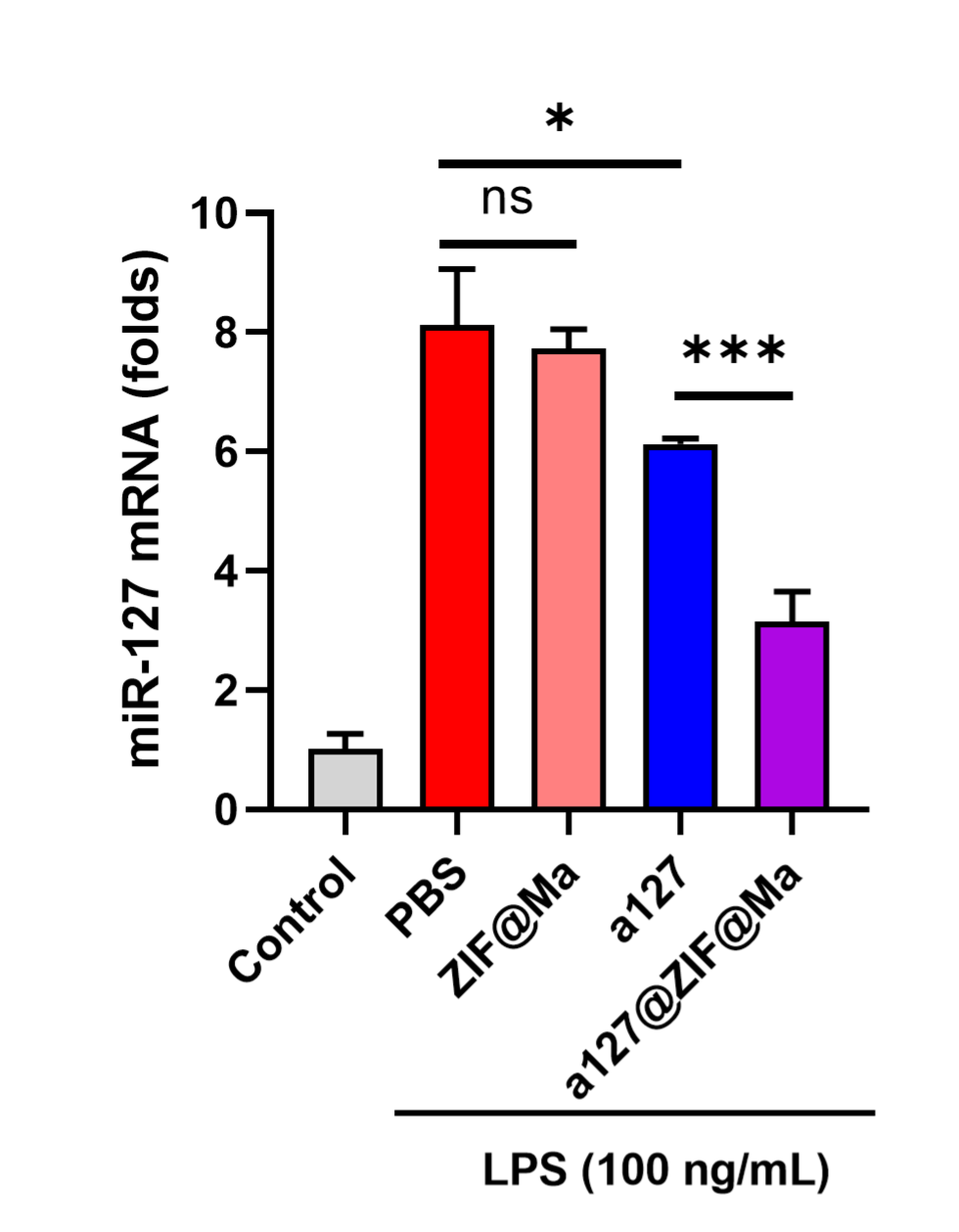


Figure S5. RAW264.7 cells were treated with ZIF@Ma, a127, a127@ZIF@Ma NPs and exposed to LPS (100 ng/mL) for an additional 12 hours apart from the control group. The mRNA expression levels of miRNA-127 were measured by qRT-PCR. All the data are expressed as the mean ± SD (n = 3); ns, no significance; *P < 0.05, **P < 0.01, ***P < 0.001.


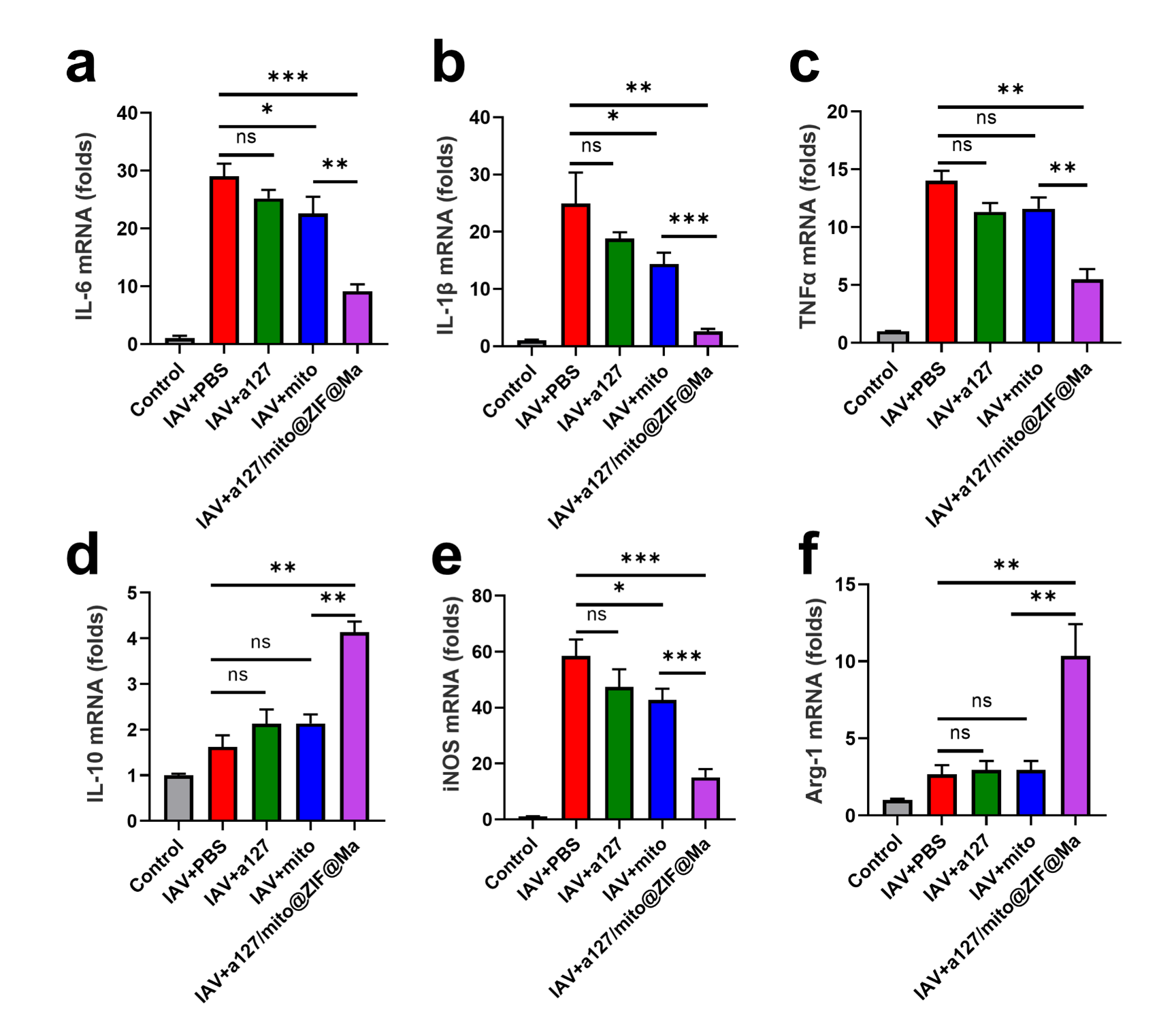


Figure S6. The therapeutic effect of a127/mito@ZIF@Ma NPs on IAV-induced acute lung injury. (a-d) The mRNA expression levels of IL-6, IL-1β, TNF-α, and IL-10 were measured by qRT-PCR. (e, f) The mRNA expression levels of iNOS and Arg-1 were measured by qRT-PCR. All the data are expressed as the mean ± SD (n = 3); ns, no significance; *P < 0.05, **P < 0.01, ***P < 0.001.


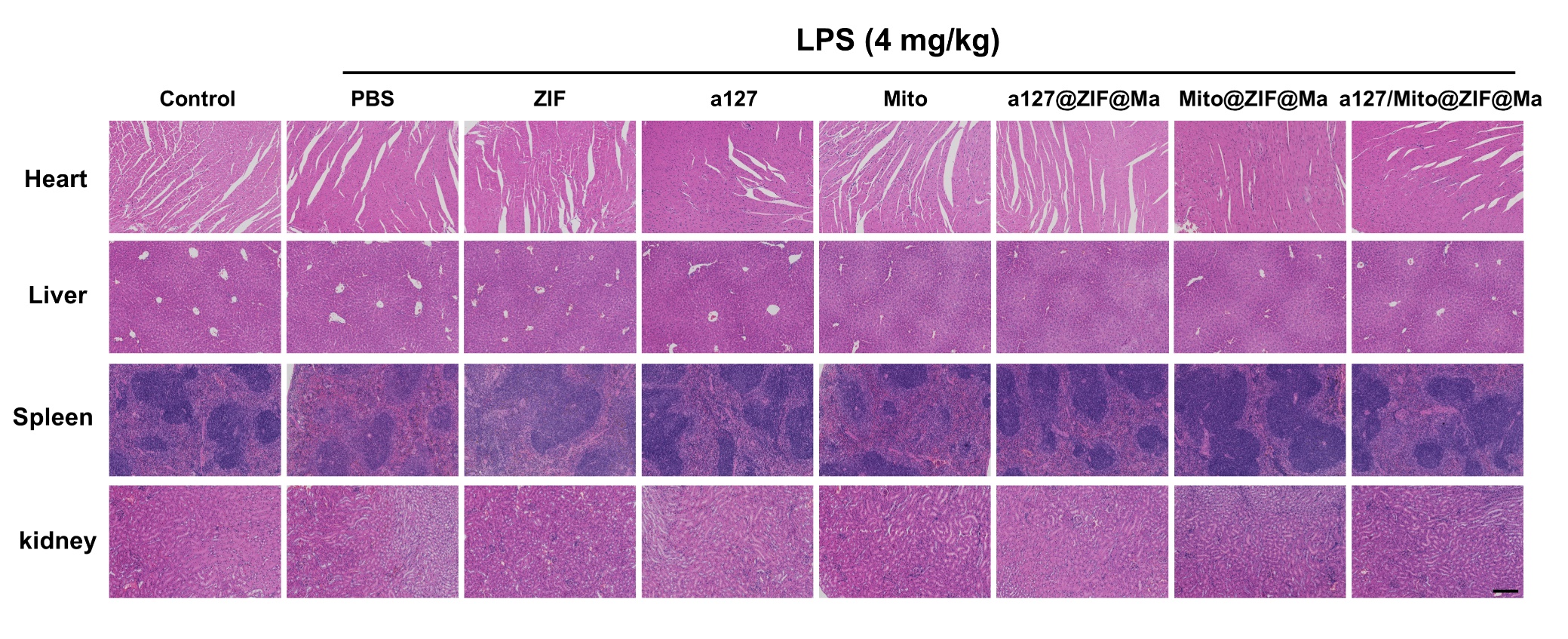


Figure S7. H&E-stained images of primary organs from mice after different treatments. All images were acquired at a magnification of 20×. Scale bar, 200 μm.


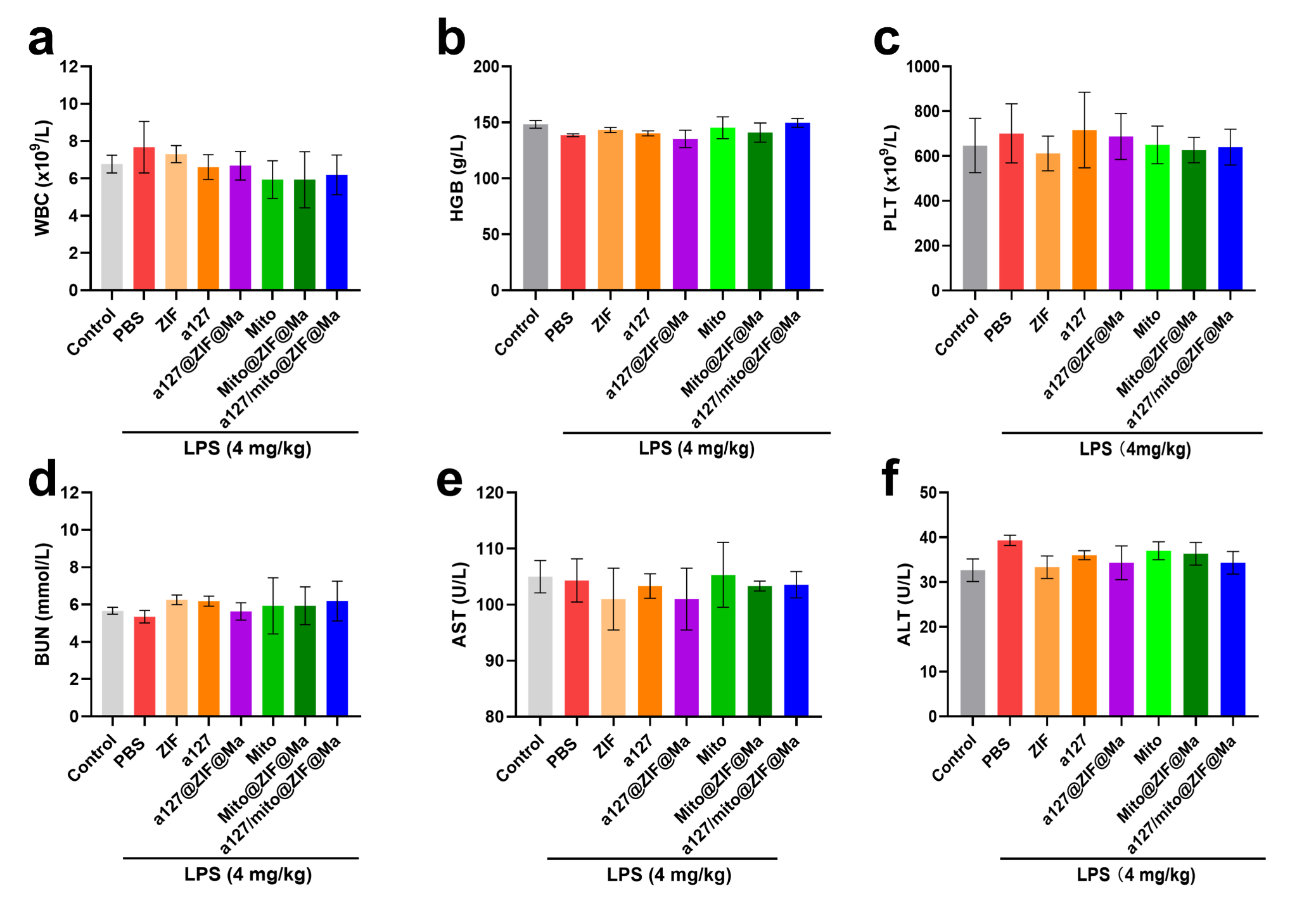


Figure S8. Serum routine analysis. (a-c) Measurement of white blood cells (WBC), glycated hemoglobin (HGB), and platelet (PLT). (d-f) Measurement of hepatic and kidney function. ALT, alanine aminotransferase; AST, aspartate aminotransferase; BUN, blood urea nitrogen. Data were presented as mean ± SD (n = 3).


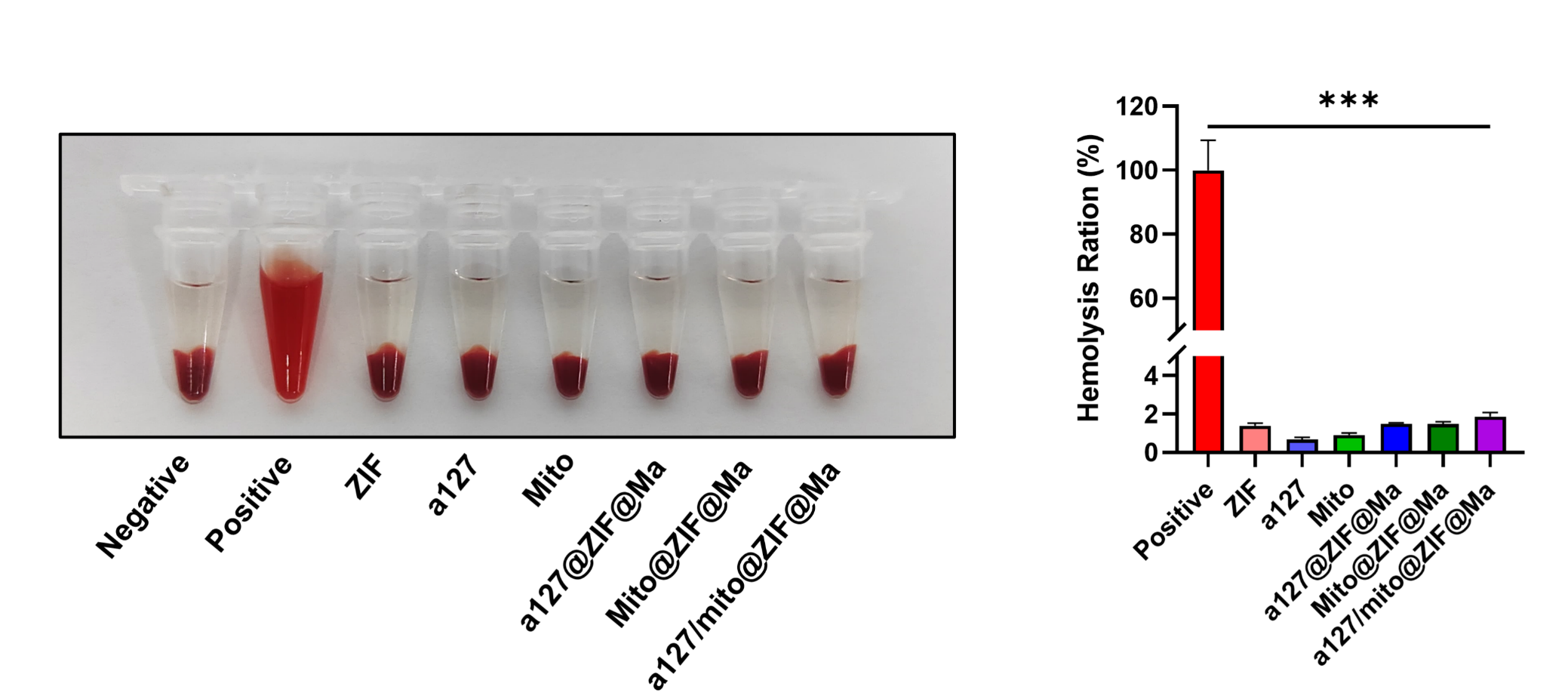


Figure S9. Image of hemolysis evaluation of ZIF-8, a127, Mito, a127@ZIF@Ma, Mito@ZIF@Ma, a127/mito@ZIF@Ma NPs and quantitative analysis of hemolysis test. ddH_2_O was served as positive and PBS was served as negative control. Data were presented as mean ± SD (n = 3). ***P < 0.001.

| Gene name | Forward primer (5’-3’ ) | Reverse primer (5’-3’ ) |
| --- | --- | --- |
| *β-actin* | CTCATGAAGATCCTGACCGAG | AGTCTAGAGCAACATAGCACAG |
| *IL-1β* | AGGCTCCGAGATGAACAA | AAGGCATTAGAAACAGTCC |
| *TNF-α* | TGTCCCTTTCACTCACTGGC | CATCTTTTGGGGGAGTGCCT |
| *IL-6* | TTCTTGGGACTGATGCTG | CTGGCTTTGTCTTTCTTGTT |
| *IL-10* | ACAGCCGGGAAGACAATAACT | GCAGCTCTAGGAGCATGTGG |
| *iNOS* | CCCTTCCGAAGTTTCTGGCAGCAGC | GGCTGTCAGAGCCTCGTGGCTTTG |
| *Arg1* | CTGGGGATTGGCAAGGTGAT | CGTTGAGTTCCGAAGCAAGC |
| *Ndnfa4* | CTGGAGCAGCACTGTATGTGA | TTGGGACCCAGTTTGTTCCAT |
| *Ndufal1* | TCCGCTTACAGCGTCTCAC | AGGCCAAACATCGCTCCAAT |
| *Ndufb3* | GAGTTTATGCTGTGCCGCTG | TACTCTGTGAAAGGCTCCGC |
| *Ndufb7* | GACCCCGAGAAGATACCCAG | GCACAGTAGTCACGTTGCTG |
| *Ndufb9* | ACCGGTACTTTGCTTGCTTG | ATCTCTCGAAGGAAGTGCCC |
| *Ndufb11* | GTCCTCCAGGGCTGTAATCG | AAAGTCAGGGTTCTTCGCGT |
| *Ndufv1* | TGCTTGTGGCTCCGACTATG | ACAGTTGTGGGGCATCCAAA |
| *Ndufv2* | GGAGGAGCCTTATTTGTGCAT | TTTGGGCGAGATCCAGGACT |
| *Sdhb* | CAGAGTCGGCCTGCAGTTT | ATCCAACACCATAGGTCCGC |
| *sdhd* | CTGGTTCCAAGGCTGCATCT | AGCCAGAGAGTAGTCCACCA |
| *Cyc1* | ATCGTTCGAGCTAGGCATGG | GCCGGGAAAGTAAGGGTTGA |
| *UgcrI1* | GGAACTGGCCAGAAACTGGA | TGCCGTTGATGTAAGGCACC |
| *Ugcrc1* | ATGCTGCGIGACATTTGCTC | TAGAAGCGCAGCCAGAACAT |
| *Uqcrq* | ATCTCCTACAGCTTGTCGCC | CTGCTCAAACTCCTGGTGC |
| *Cox5a* | TGTCTGTTCCATTCGCTGCT | AACCGTCTACATGCTCGCAA |
| *Cox5b* | GCTTCAAGGTTACTTCGCGG | ATGGGTCCAGTCCCTTCTGT |
| *Cox6a1* | CAACGTGTTCCTCAAGTCGC | CTTCATAGCCGGTCGGAAGT |
| *Cox6b1* | AGAACTACAAAACTGCCCCCT | TTCTCACAGCGGTGGAAGTC |
| *Cox7c* | GAGTATCCGGAGGTTCACGAC | ACCGCCACTTGTTTTCCACT |
| *Cox8a* | CAGGTCCACTCGAAGCCG | CACGCAGAAGACAACACACG |
| *ATP5d* | TACGCTGACTGGAGCCTTTG | GTCCAGCATGTCCAGTGTCA |
| *ATP5e* | TCAGCTACATCCGGTTTTCCC | TTTTATGCTGCTGCCCGAAG |
| *ATP5g2* | ATGTACGCCTGCTCCAAGTT | CTGTGGTCGCTTCAACTCCA |
| *ATP6V1* | ACATCGCAGAGATGGTTCGG | CTLTGGCTGCATCGTAGGGA |
| *ATP6v0c* | GTCCCGTTGTCCTAGCTCGC | TCCTAGAAGCTGGGTGCAGAA |
| *ATP5h* | TGGAATGAGACCTTCCACGC | GCACAGGAATCTTCAGGGCAG |

Table 1. Primer sequences for quantitative RT-PCR analysis.
